# Dispersal and Diving Adjustments of the Green Turtle *Chelonia mydas* in Response to Dynamic Environmental Conditions during Post-Nesting Migration

**DOI:** 10.1371/journal.pone.0137340

**Published:** 2015-09-23

**Authors:** Philippine Chambault, David Pinaud, Vincent Vantrepotte, Laurent Kelle, Mathieu Entraygues, Christophe Guinet, Rachel Berzins, Karin Bilo, Philippe Gaspar, Benoît de Thoisy, Yvon Le Maho, Damien Chevallier

**Affiliations:** 1 Université de Strasbourg, Institut Pluridisciplinaire Hubert Curien, 23 rue Becquerel, F-67087 Strasbourg cedex 2, France; 2 CNRS, UMR 7178, 23 rue Becquerel, F-67087 Strasbourg cedex 2, France; 3 Centre d’Études Biologiques de Chizé, UMR 7372 CNRS—Université de La Rochelle, 79360 Villiers-en-Bois, France; 4 Laboratoire d’Océanologie et de Géosciences, UMR 8187 CNRS, 28 avenue Foch, BP 80 62930 Wimereux, France; 5 CNRS Guyane, USR 3456, av. Charlery, 97300 Cayenne, France; 6 WWF Guyane, N°5 Lotissement Katoury, F-97300 Cayenne, France; 7 Office National de la Chasse et de la Faune Sauvage—Cellule technique Guyane, Campus agronomique, BP 316, 97379 Kourou cedex, France; 8 WWF Guianas, Henck Arronstraat 63, Paramaribo, Suriname; 9 Collecte Localisation Satellites, Direction Océanographie Spatiale, 8–10 rue Hermès, 31520 Ramonville, France; 10 Association Kwata, 16 avenue Pasteur, BP 672, F-97335 Cayenne cedex, France; Sonoma State University, UNITED STATES

## Abstract

In response to seasonality and spatial segregation of resources, sea turtles undertake long journeys between their nesting sites and foraging grounds. While satellite tracking has made it possible to outline their migration routes, we still have little knowledge of how they select their foraging grounds and adapt their migration to dynamic environmental conditions. Here, we analyzed the trajectories and diving behavior of 19 adult green turtles (*Chelonia mydas*) during their post-nesting migration from French Guiana and Suriname to their foraging grounds off the coast of Brazil. First Passage Time analysis was used to identify foraging areas located off Ceará state of Brazil, where the associated habitat corresponds to favorable conditions for seagrass growth, i.e. clear and shallow waters. The dispersal and diving patterns of the turtles revealed several behavioral adaptations to the strong hydrodynamic processes induced by both the North Brazil current and the Amazon River plume. All green turtles migrated south-eastward after the nesting season, confirming that they coped with the strong counter North Brazil current by using a tight corridor close to the shore. The time spent within the Amazon plume also altered the location of their feeding habitats as the longer individuals stayed within the plume, the sooner they initiated foraging. The green turtles performed deeper and shorter dives while crossing the mouth of the Amazon, a strategy which would help turtles avoid the most turbulent upper surface layers of the plume. These adjustments reveal the remarkable plasticity of this green turtle population when reducing energy costs induced by migration.

## Introduction

Seasonality drives the availability and aggregation of resources, and is thus one of the main ecological factors affecting the evolution and the ecology of long-distance migrants [1]. Indeed, in most cases, the requirements of migratory animals temporally and spatially match the peak of resource abundance, thus avoiding resource depletion. Migrant organisms time their movements according to their life stages and their different activities (growth, breeding, etc.) in order to exploit seasonal resources that vary at temporal and spatial scales, generally travelling long distances to reach appropriate sites for their needs [2]. It is crucial to assess the dispersal movements and the habitat used by migrating animals in order to understand their ecology and facilitate the implementation of adequate conservation policies [3]. This migratory behavior has been studied in a wide range of marine groups such as mammals [4,5], birds [6], fish [7,8] and reptiles [9–11].

Sea turtles are long-distance migrants that undertake long journeys from their nesting sites to foraging grounds [12]. Most nesting sites do not provide sufficient energy resources for turtles to sustain oviposition and year-round residency [10]. Additionally, the turtle allocates the majority of its energy to reproduction during breeding and nesting, resulting in high energy requirements at the end of the nesting season [13,14]. The turtles therefore migrate after the nesting season to replenish their body reserves, foraging in areas of high productivity in order to maximize their foraging efficiency.

The migratory strategy associated with specific foraging grounds varies greatly across sea turtle populations [15]. Most of adult Cheloniidae, i.e. hawksbill, loggerhead, and green turtles, usually migrate across the open ocean to reach neritic feeding grounds [16]. However, while satellite tracking has made it possible to outline the migration routes, we still have little knowledge of how sea turtles select their foraging grounds [17].

Adult Cheloniidae sea turtles feed on different resources, depending among others on their ecological requirements, their diet and on the habitat characteristics, i.e. resource availability, competition, etc. Unlike the omnivorous (olive ridley and hawksbill) and carnivorous (loggerhead) species of sea turtles [18], the green turtle *Chelonia mydas* is mainly a herbivorous grazer at the adult stage, and is therefore dependent on seagrasses or algae meadows [18]. A high diversity of seagrass species [19] can be found throughout the western part of the Tropical Atlantic from the Gulf of Mexico to the north part of the Brazilian coast (up to 10°S). These marine meadows provide foraging grounds for several green turtle populations [20] originating from seven different rookeries: Ascension Island, Matapica (Suriname), Aves Island (Venezuela), X’cacel and Isla Cozumel (Mexico), Tortuguero (Costa Rica) and the east coast of Florida in the United States [20].

In addition to these seven populations, there is another little-known rookery in the western Equatorial Atlantic, located at the natural border between French Guiana and Suriname along the beaches of the Maroni estuary [21]. Since seagrass is distributed throughout the Tropical Atlantic [19], there is a possibility that turtles from this nesting site could either migrate north-westward, following the Guiana current flow to reach high density seagrass beds found in the Caribbean, or swim along the Brazilian coasts further south [19,22]. Baudouin et al. (2015) found that they undertake a south-eastward migration, presumably swimming against the strong North Brazil current and crossing an unfavorable and highly turbulent zone, the Amazon River plume.

The Amazon River is a major source of freshwater input, supplying 20% of the freshwater entering the ocean [23]. It has the highest level of water and sediment discharge and the largest drainage basin in the world [23–25]. The Amazon plume discharges 115.10^7^ tons of sediments into the Equatorial Atlantic Ocean per year, strongly influencing the oceanographic and biochemical processes of the north-eastern American coast [24–26]. The large amounts of suspended materials carried by the plume lead to low levels of irradiance, hampering phytoplankton photosynthesis [27]. *A priori*, such turbid waters are therefore an unsuitable foraging habitat for herbivorous organisms [28].

If green turtles cross this particularly turbulent zone as part of their migratory strategy, there must be a reason for undertaking this long and counter-current migration to reach specific areas off the Brazilian coast. Our study is based on satellite telemetry, and attempts to shed light on how green turtles nesting in French Guiana and Suriname select their foraging grounds and adapt their post-nesting migration to dynamic environmental conditions. Our two main objectives in this study of green turtle migration strategy were therefore (i) to locate the foraging grounds and characterize habitat affinities and (ii) to assess how oceanographic conditions encountered along the way can affect the movements of turtles in terms of dispersal and diving patterns.

## Materials and Methods

### Ethics statements

This study meets the legal requirements of the countries where this work was carried out, and follows all institutional guidelines. The protocol was approved by the “Conseil National de la Protection de la Nature” (CNPN, http://www.conservation-nature.fr/acteurs2.php?id=11), a branch of the French ministry for ecology, sustainable development and energy (permit Number: 09/618) acting as an ethics committee in French Guiana and Suriname. After the evaluation of the project by the CNPN, fieldwork was carried out in strict accordance with the recommendations of the Police Prefecture of French Guiana, Cayenne, France, in order to minimize the disturbance of animals.

### Satellite tag deployment

During the inter-nesting season, 16 Argos-linked Fastloc GPS tags (MK10, Wildlife Computers Redmond, WA, USA) were deployed on adult female green turtles from February to June 2012 on both sides of the Maroni River: at Awala-Yalimapo in the Amana Nature Reserve, French Guiana (5.7°N-53.9°W, *n* = 8), and in the Galibi Nature Reserve in Suriname (5.4°N-53.5°W, *n* = 8) [21]. During the same period in 2014, 10 additional females in the Amana Nature Reserve were equipped with Conductivity Temperature Depth-Fluorometer Satellite Relayed Data Loggers (CTD-SRDL, Sea Mammal Research Unit, University of St. Andrews, Scotland). The attachment procedure followed the standard methods described in Baudouin et al. [21]. During tag deployment, measurements of the Curved Carapace Length (CCL) were taken, and body mass could then be calculated using Hays et al.’s method [29]–see S1 Table.

### Data collected

The procedure to extract migratory route data was identical to that used in Baudouin et al. [21]. The Argos-linked Fastloc GPS tags also provided diving data, i.e. maximum dive depths, dive durations and *in situ* temperature data, binned as 4-hour period histograms. Maximum depths were collected in different bins, every 10 m from 10 to 100 m, then every 50 m from 100 to 250 m. Maximum dive durations were stored from 30 s to 1 min, then every minute from 1 to 5 min, and finally every 10 minutes from 10 to 60 min. *In situ* temperatures were recorded during dives from 20 to 32°C, every one degree Celsius. Tags also supplied Time At Depth (TAD) and Time At Temperature (TAT), defined as the proportion of time (in %) spent at each depth and the temperature range, respectively.

The CTD-SRDL tags provided the locations of animals, i.e. Argos data, and simplified profiles of the diving parameters, and oceanographic data. However, the oceanographic and diving data were not used in the analysis.

### Data pre-filtering

In order to retain only the positions recorded during the migration, a spatial query was performed via ArcGIS version 10.1 to identify the date migration began. By calculating the average daily speed during the inter-nesting season, a speed filter of 30 km.d^-1^ was set, and only the positions associated with a daily speed > 30 km.d^-1^ were set to migration phase and then retained for the analysis.

Using the same approach as described in Heerah et al. [30], a Kalman-filtering algorithm was then applied (*CLS*, *Collecte Localisation Satellites*, Toulouse, France) to enhance tag position estimates (Argos and GPS) by accounting for Argos location errors [31,32]. The shoreline was extracted from NOAA National Geophysical Data Center, Coastline, e.g. WVS, GSHHG. The General Bathymetric Chart of the Oceans database (GEBCO, http://www.gebco.net/, 30-arc-second 1 km grid) was used to discard any locations on land. The positions associated with a speed of over 10 km.h^-1^ and those with location class Z (0.1%, class associated with the raw location before Kalman filtering) were also removed, considered insufficiently accurate, and any dive depth records from tags over 100 m were also removed due to the substantial differences between the depth values provided by the pressure sensors of the tags and bathymetry data. Seven individuals (#115451, #115453, #130766, #130769, #130776, #131354 and #131355) were discarded from the analysis due to insufficient data caused by transmission issues.

### First Passage Time analysis

After proceeding with pre-filtering, First Passage Time (FPT) analysis was performed on location data (Argos location after kalman filtering and GPS) in order to spatially and temporally identify Areas of Restricted Search (ARS) using R software version 3.2.1 [33]. FPT is defined as the time required by an organism to cross a circle of a given radius. FPT approach is a three-step procedure:

1. The track of each turtle was linearly interpolated at 1 km intervals whilst retaining raw locations to avoid losing data.
2. FPTs were then calculated at every location of the interpolated tracks for radii ranging from 1 to 400 km to ensure the coverage of large foraging movements [34]. For each track, the relative variance of FPT (after log transformation) was plotted against radii to identify the scales of searching activity (ARS) revealed by a peak of variance at a specific radius (S1a Fig). If several peaks appeared, we only considered the peak corresponding to maximum variance, as the study focuses on the smallest foraging scale.
3. Finally, by plotting the FPT at the optimal scale as a function of time, the periods featuring ARS (higher FPT) could be identified throughout the trip [35–37]–see S1b Fig. Temporal detection of ARS periods was carried out using Lavielle’s segmentation method [38] from the *adehabitatLT* package, which allowed to differentiate between twomodes: No ARS (low FPT, transiting mode) vs. ARS (high FPT, foraging mode). The migratory mode of turtles (No ARS vs. ARS) was then inferred to each position after identifying temporal ARS locations from FPT outputs (S1b Fig).

### Track segmentation

When considering the transiting mode only, the trajectories were delineated into three phases to take into account the influence of the Amazon River plume on the horizontal and vertical movements of turtles (Phase 1: before plume, Phase 2: within plume and Phase 3: after plume). Along the tracks of the turtles, the trajectories were segmented based on the distribution of the diffuse attenuation coefficient at 490 nm (hereafter called K_d_ in m^-1^ –see S2 Fig). The K_d_ 490 nm is a standard ocean color product of downwelling irradiance at 490 nm, operationally provided by the various ocean color sensors, and considered as a good index for qualifying the light attenuation of the visible light in the water column. Averaged monthly data for K_d_, an indicator of the turbidity of the water column, were extracted at a 4 km resolution from the Ocean Color website (http://oceancolor.gsfc.nasa.gov/).

### Spatial analysis

For the spatial analysis, the distance travelled and the elapsed time between locations were calculated using the *trackDistance* function from the *trip* package on R [39]. The observed speed was then derived from the distance and time elapsed between locations. The distance to shore was also calculated within each of the three phases for the 19 individuals retained for the study. To investigate the role of oceanic circulation on turtle movements, surface current data (meridional and zonal components) were extracted daily for the tracking period from Mercator-Ocean GLORYS-2v1 (Global Ocean ReanalYsis and Simulations, available on: http://marine.copernicus.eu/web/69-myocean-interactive-catalogue.php) model, at a 0.25° resolution (~28 km). Oceanic current velocity and the associated direction were then derived from meridional and zonal components (scalars u and v respectively). The average swimming speed of the turtles was then calculated with correction for current velocity following Gaspar et al.’s method [40], giving a proxy of the swimming effort.

The time taken by each turtle to reach the foraging grounds (hereafter called FG) was derived from FPT outputs (S1 Fig). To assess the effects of the plume on the overall migration, we performed linear models using the time spent within the plume (TimePhase2) as a response variable, using the *lm* function from the *stat* package on R. Since the significantly lower number of locations recorded within the plume did not permit the precise calculation of the observed speed, we decided to use duration within the plume instead of the travel speed. We therefore selected one *temporal variable*, i.e. the time taken to reach a foraging ground, one *spatial variable*, i.e. the minimum longitude of the foraging ground (location of the closest foraging site reached in relation to the nesting site), one *variable relative to the movement of turtles*, i.e. the averaged swimming speed of turtles within the plume, and one *intrinsic covariate*, i.e. the body mass. Indeed, given the strong hydrodynamic forcing generated by the Amazon River plume, it can be assumed that the time taken to reach the foraging ground will increase with increasing time within the plume. In addition, we assumed that the first turtles that stop to forage, i.e. at the closest recorded foraging ground in relation to the nesting site, would have spent more time crossing the plume. Finally, we assumed that the time within the plume would increase with higher current velocity, and, conversely, would decrease with increased swimming speed. Following the method described in Zuur et al. [41], all combinations of collinear predictors (Spearman coefficients > 0.8 and < −0.8) were excluded, then all possible combinations without interactions were tested and the model with the lowest Akaike Information Criteria (AIC) was selected.

### Habitat affinities

To characterize the foraging habitat in relation to environmental conditions, four static and dynamic remotely sensed variables were selected according to their biological relevance and availability in the study area. We extracted bathymetry data from GEBCO, then used the *terrain* function from the *raster* package [42] to derive the slope and obtain an indicator of seabed roughness. Two oceanographic variables were also extracted from Ocean Color website, namely K_d_ and Sea Surface Temperature (SST, 11 micron per day, http://oceancolor.gsfc.nasa.gov/cms/). The monthly data provided were already averaged at a 4 km resolution. A full coverage of the satellite remote sensing data over the whole area of interest could not be obtained, mostly due to cloud coverage or failures of the atmospheric correction procedure. Missing satellite data were therefore estimated by interpolating the SST and K_d_ using inverse distance weighting method from *gstat* and *raster* packages [42,43]. The covariate values were then extracted at the locations of each turtle (the GPS and Kalman filtered Argos locations) using the *extract* function from the raster package [42].

### Diving behavior analysis

For the Argos-linked Fastloc GPS tags, no precise location was associated with each dive as diving data were stored in 4h-histograms. FPT could not therefore be applied to these data. To differentiate the diving data between the two behavioral modes, i.e. transiting vs. foraging, we therefore relied on the starting date of the foraging behavior, as identified by FPT analysis. The position data obtained from the diving records were then segmented into two modes, i.e. transiting vs. foraging. To assess the effect of the plume on diving behavior during the transiting mode, the positions were also segmented into three phases based on the K_d_ distribution, i.e. before plume, within plume and after plume. As the CTD-SRDL tags provided a very small amount of diving data, only data provided by the Argos-linked Fastloc GPS tags were included in the diving behavior analysis.

### Statistical analyses

All statistical analyses were performed using R software version 3.2.1 [33]. All samples submitted to statistical tests were first checked for normality and homogeneity of variances by means using Shapiro-Wilk test. Depending on the results, parametric or nonparametric tests were used. Globally, Wilcoxon-Mann-Whitney tests were used to compare the diving behavior and the environmental variables between the two modes, i.e. transiting vs. foraging, using a significance level of α = 0.05. Tukey HSD tests were used to compare the behavior between the three migration phases. Values are means ± SD.

## Results

### Foraging grounds selection

#### Foraging ground locations and spatial scales

**Fig 1:**
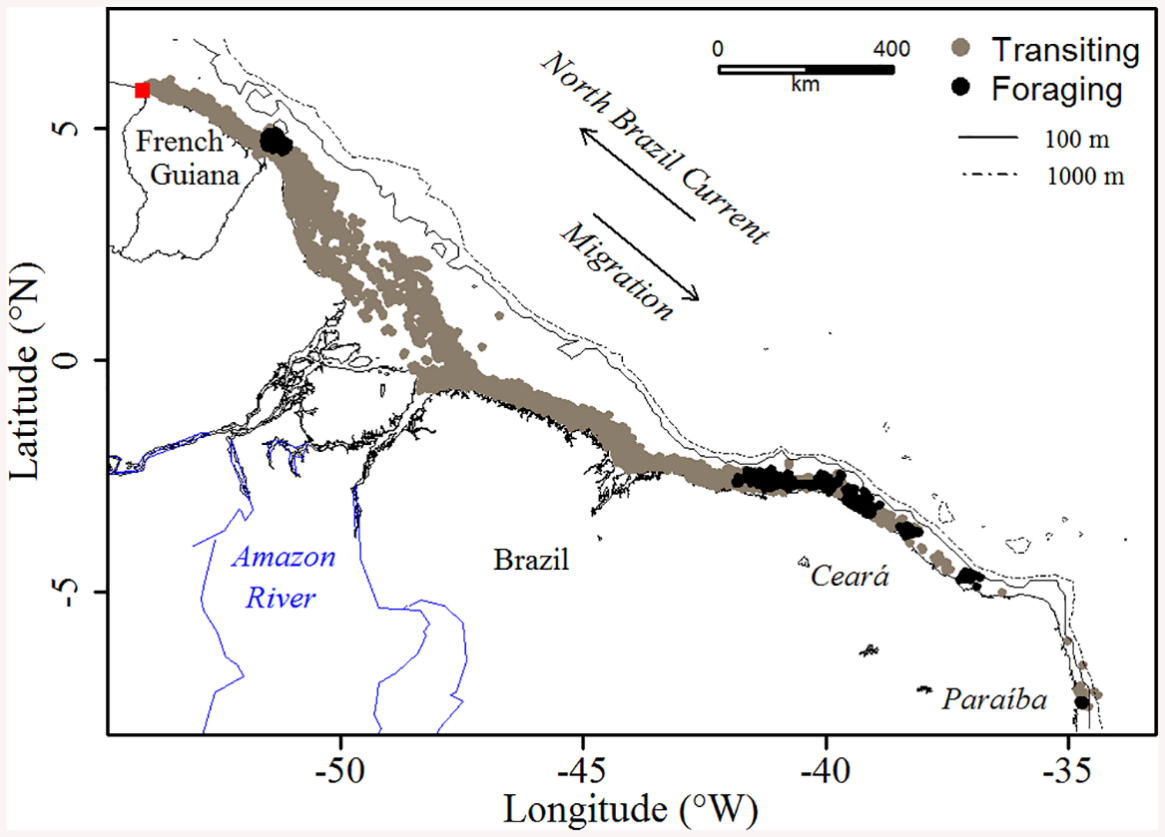
Locations of the 19 green turtles equipped in 2012 and 2014 for the two behavioral modes, i.e. transiting (gray) and foraging (black). The red square indicates the migration departure point. The shoreline was extracted from NOAA National Geophysical Data Center, Coastline.

For both years of tag deployment, the 19 analyzed tracks of *Chelonia mydas* showed a south-eastward migration over an average 3450±701 km for a mean tracking duration of 118±37 days (Fig 1 and S1 Table). All turtles used a narrow corridor (mean: 22.5±24.4 km), remained on the continental shelf (< 100 m isobaths) and migrated at an average observed speed of 0.5±0.5 m.s^-1^ (~2 km.h^-1^)–see Table 1.

**Table 1:** Summary of the horizontal and vertical movements of the 19 green turtles during the three migration phases. The diving parameters refer only to the 14 Argos-Fastloc GPS tags. The numbers 1, 2 and 3 refer to the three migration phases, respectively before, within and after the plume.

	Migration phase			All migration
	1	2	3	Mean±SD
Distance to shore (km)	13.5±8.4	46.6±45.7	17.3±11.3	22.5±24.4
Observed speed (m.s^-1^)	0.6±0.5	0.8±0.6	0.6±0.5	0.5±0.5
Swimming speed (m.s^-1^)	1.0±0.5	1.1±0.6	0.9±0.5	0.8±0.5
Current velocity (m.s^-1^)	0.5±0.2	0.4±0.3	0.3±0.1	0.4±0.2
Maximum depth (m)	26.6±25.3	38.7±28.8	34.5±18.4	32.0±20.9
Maximum duration (min)	35.2±23.6	29.1±23.4	37.4±22.5	35.1±21.9

FPT analysis was used to locate the foraging areas both in time and space (S1 Fig). With the exception of one turtle that stopped off the coast of Cayenne in 2012 (#115446, foraging event in Phase 1) rather than at the very end of turtle tracks, the searching activity recorded spans from June to mid-September, and foraging areas were found to be located at the very end of the turtle tracks, along the shores of Ceará and Paraíba states, Brazil (Fig 1 and S1 Table). The radii of the foraging areas ranged from 7 to 60 km (#115448 vs. #115446), with an average of 19.7±14.6 km over the whole foraging trip. Twenty-one percent of the turtles used fine-scale foraging (<10 km radius, #115448, #115456, #115458 and #115460), 47% foraged on a medium-scale (10–20 km, #115445, #115449, #115450, #115452, #115454, #115455, #115457, #130767 and #130773), and 21% showed coarser-scale foraging beyond a radius of 20 km (#115446, #115447, #115459 and #130770), while First Passage Time analysis revealed a complete absence of foraging behavior in two individuals (#130768 and #130771) (S1 Table).

#### Habitat affinities

**Fig 2:**
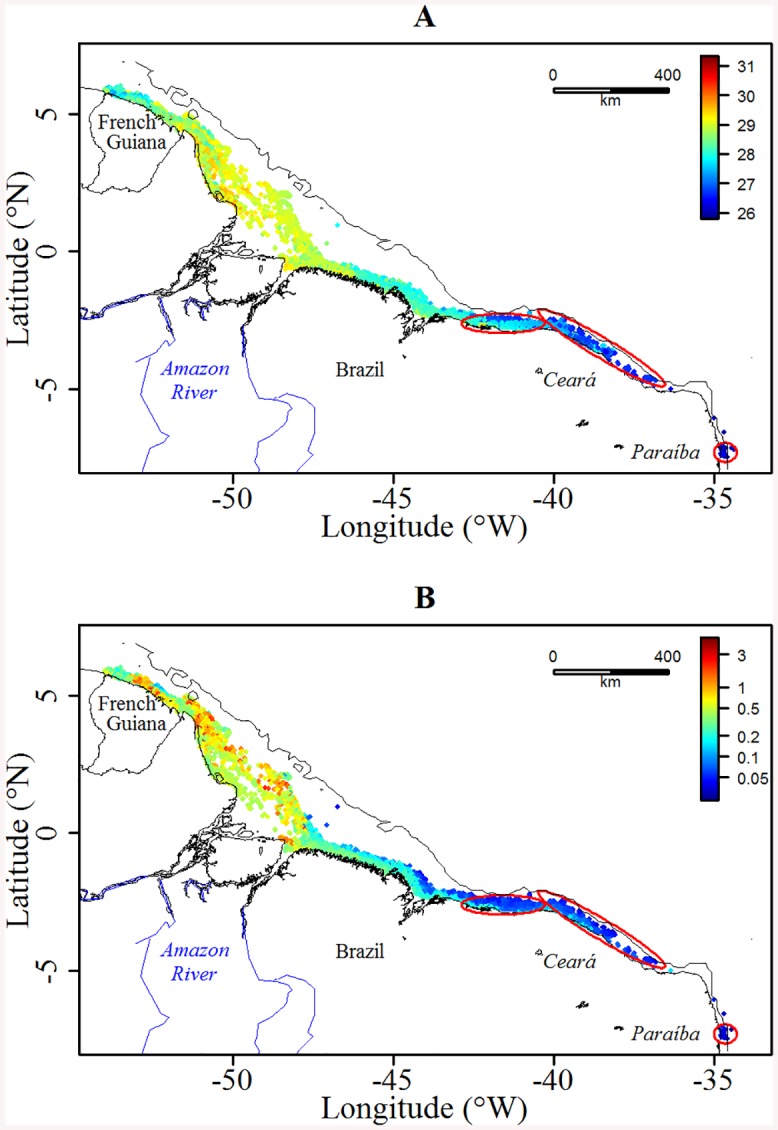
Distributions of (A) SST (°C) and (B) K_d_ (m^-1^) along the 19 turtles’ tracks. The foraging grounds are represented by the red ellipses and the black solid line refers to the 100 m isobaths. K_d_ refers to the Diffuse Attenuation Coefficient and was logged transformed for a better contrast. The shoreline was extracted from NOAA National Geophysical Data Center, Coastline.

**Fig 3:**
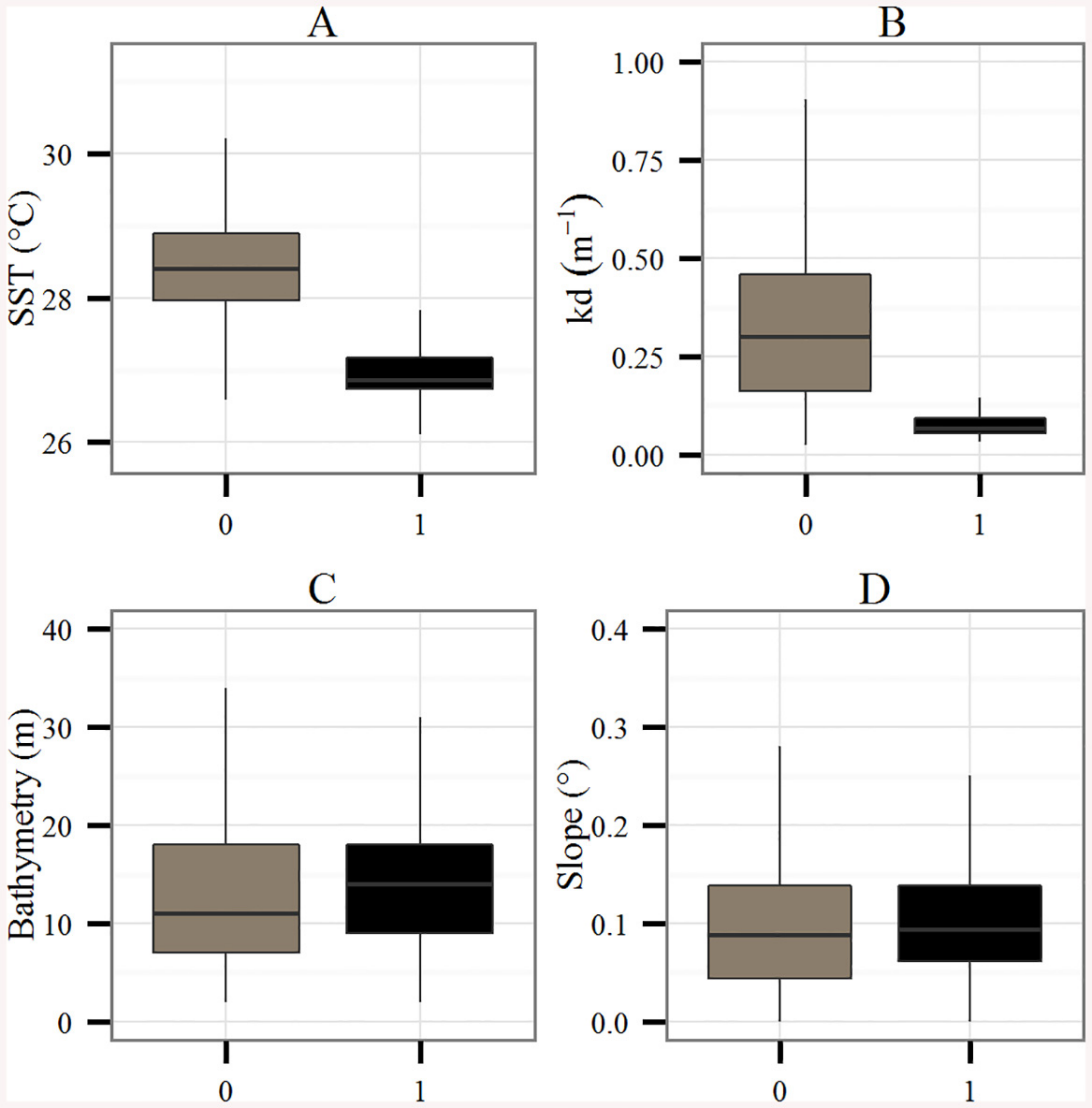
Box plots of (A) the SST (°C), (B) the K_d_ (m^-1^), (C) the Bathymetry (m) and (D) the Slope (°) extracted at turtles’ locations for the two behavioral modes, i.e. transiting (grey) and foraging (black).

During their migration, green turtles crossed highly contrasted environmental conditions (Fig 2). In the foraging mode, the SST was significantly lower than in the transiting mode (27.0±0.4 vs. 28.3±0.6°C, respectively, Wilcoxon test *p* < 0.001)–see Figs 2A and 3A. Identically, the K_d_ was significantly lower at the foraging grounds (0.09±0.09 vs. 0.41±0.38 m^-1^, respectively, Wilcoxon test *p* < 0.001, Figs 2B and 3B). In contrast, the bathymetry was slightly deeper outside the feeding grounds (15.2±31.9 vs. 14.2±9.2 m, respectively, Wilcoxon test *p* < 0.001, Fig 3C). The slope remained weak over the whole migration but was slightly steeper at the foraging grounds (0.13±0.28 vs. 0.12±0.10 m, respectively, Wilcoxon test *p* < 0.05, Fig 3D).

#### Diving behavior

**Fig 4:**
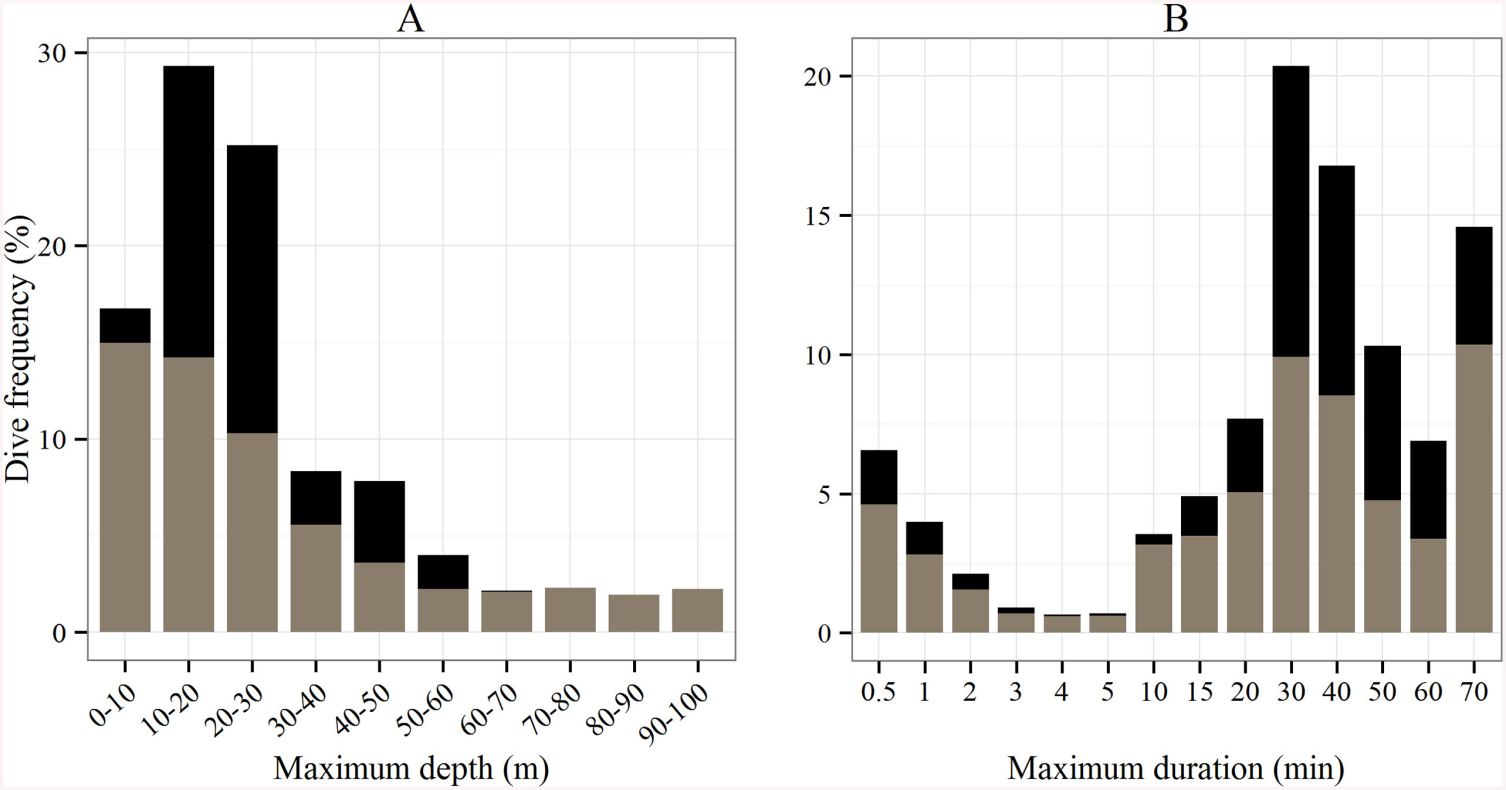
Histograms of (A) the maximum dive depth (m) and (B) the maximum dive duration (min) recorded by the 14 Argos-Fastloc GPS tags for the two behavioral modes, i.e. transiting (gray) and foraging (black).

Among the 974 dive depths recorded, 1% were discarded due to biologically implausible depth records when comparing the depth recorded by the tag to the bathymetry for the same location (>100 m). When considering all tracks as a whole, the maximum dive depth ranged from 10 to 100 m, and 70% of the dives were performed at shallow depths within 30 m of the surface (Fig 4A). The depth range was greater outside the foraging areas and ranged from 10 to 100 m, whereas turtles concentrated their dives between 10–30 m at the foraging grounds. Mean depth was significantly higher outside the foraging areas (Wilcoxon test: *p* < 0.001, 29.5±12 m vs. 33.7±25.1 m)–see Fig 4A.

Regarding the overall distribution of the maximum dive duration, the dives ranged from 0.5 to 70 min and 35% of the dives lasted 30–40 min (Fig 4B). Wherever the foraging area was located, i.e. whether it was off the shores of Cayenne or at the end of the migratory path, dives lasted significantly longer at the foraging grounds (37.3±19.1 min) than during the transiting between feeding sites (33.6±23.5 min, Wilcoxon test: *p* < 0.001).

### Behavioral adaptations to environmental conditions

#### Distance to shore

**Fig 5:**
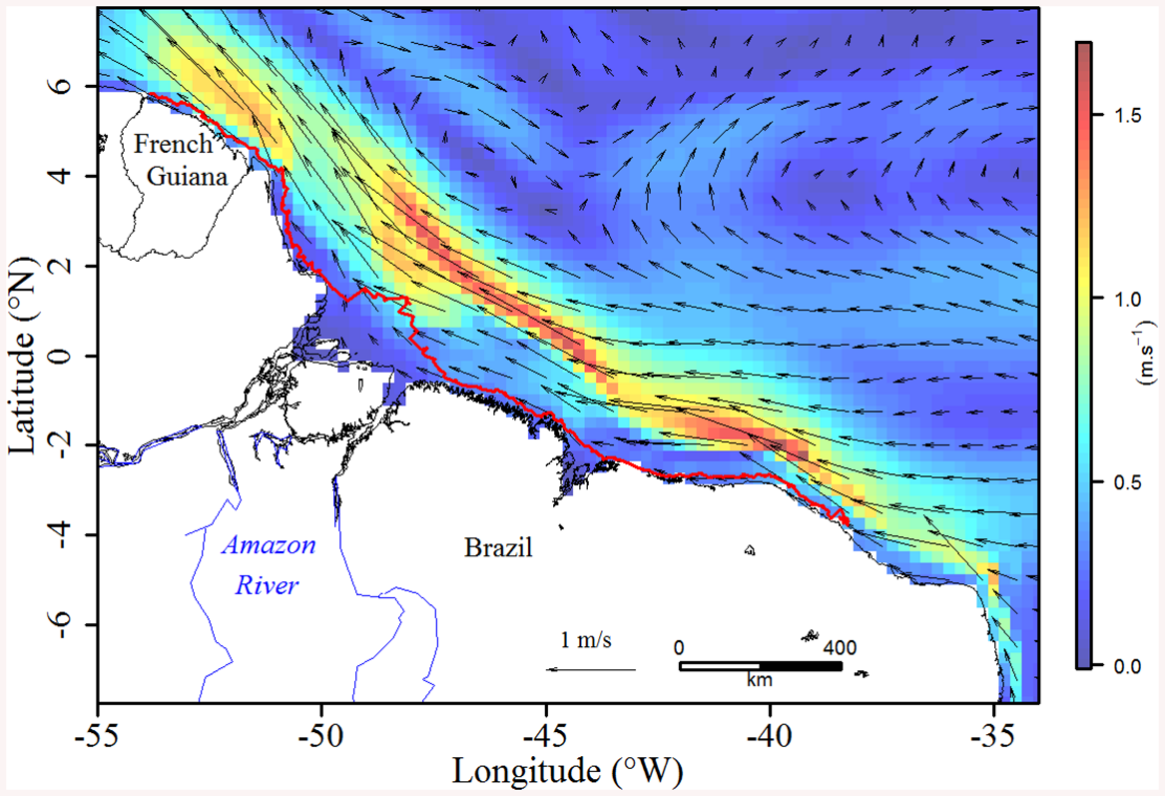
Mean direction and velocity of the currents over the whole study area for the 2012 and 2014 tracking periods extracted daily from Mercator-Ocean GLORYS-2v1 (Global Ocean ReanalYsis and Simulations). The trajectory of turtle #115452 (red) is superimposed on the current. For a better visual representation, the spatial resolution of the current direction is set to 0.75 degrees. The shoreline was extracted from NOAA National Geophysical Data Center, Coastline.

Over the entire migration, 88% of the locations were located in areas with a north-westward current flowing against the migratory path of the turtles (Fig 5). However, the strongest velocities flow parallel to the coastline at around 100 km offshore, i.e. the North Brazil current, and turtles remained on average within 22.5±24.4 km of the shore (Table 1). Current velocity was positively correlated to the distance to the shore (Spearman correlation test: R^2^ = 0.36, *p* < 0.001), especially within the plume (Spearman correlation test: R^2^ = 0.57, *p* < 0.001).

**Fig 6:**
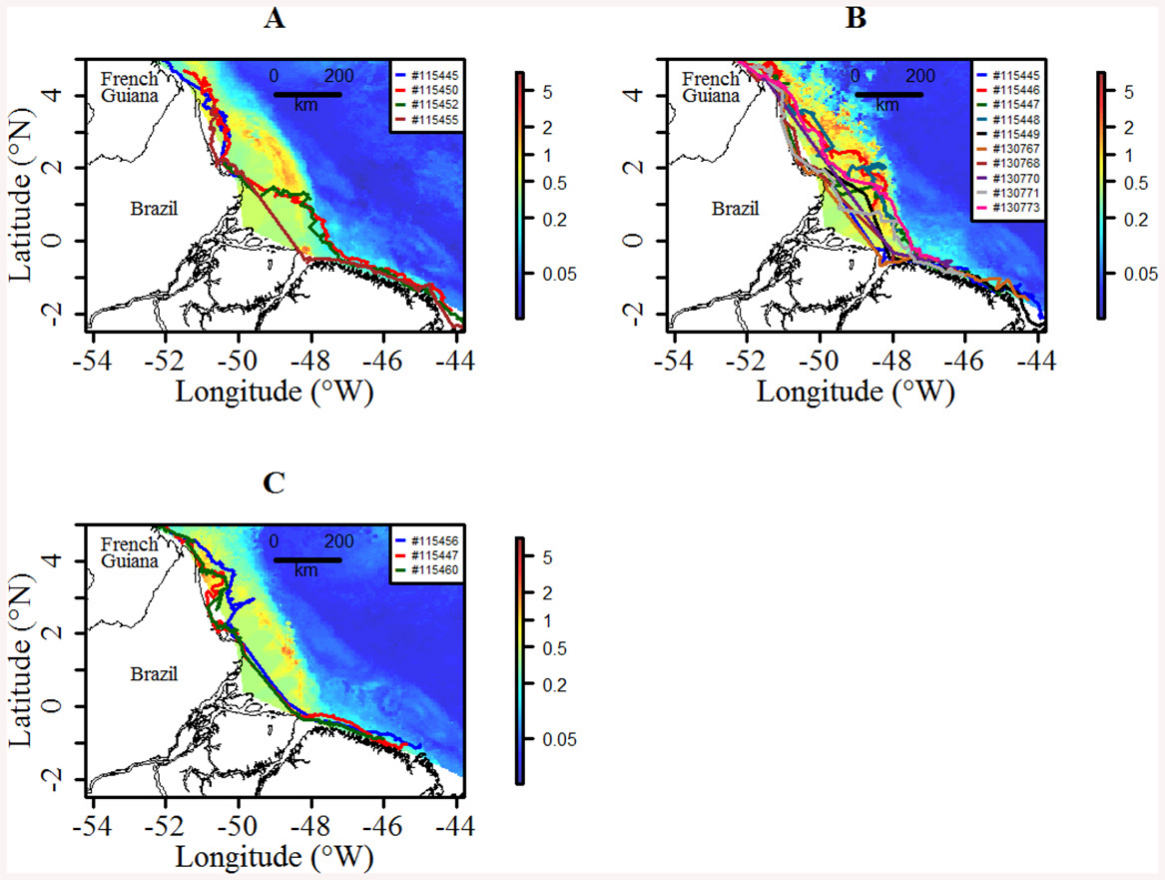
Map of K_d_ (m^-1^) within the plume for (A) May, (B) June and (C) July, with turtle routes superimposed for the corresponding months. The K_d_ was log transformed to improve representation and extracted monthly from Ocean Colour database and the shoreline comes from NOAA National Geophysical Data Center, Coastline.

The distance to shore varied significantly between all migration phases (Tukey HSD: *p* < 0.001, Table 1). Turtles swam closer to shore before and after the mouth of the Amazon (Phases 1 and 3), swimming over twice as far from the coast while crossing the plume (46.6±45.7 km) than in any other parts (17.4±11.4 km, Fig 6).

#### Speed

Except between Phase 1 and Phase 3, the observed speed differed significantly according to the migration phase, and was highest within the plume and lowest before and after the plume (0.8±0.6 m.s^-1^ vs. 0.6±0.5 m.s^-1^, Tukey HSD: *p* < 0.01, Table 1). Over the entire migration, the average swimming speed after correction for currents was higher than the observed speed (0.5±0.5 m.s^-1^ vs. 0.8±0.5 m.s^-1^ ~3 km.h^-1^), with minimum values observed after the plume and maximum values recorded at the Amazon mouth in Phase 2 (0.9±0.5 m.s^-1^ vs. 1.1±0.6 m.s^-1^). There was also a positive relationship between the current velocity and the swimming speed (Spearman correlation test: R^2^ = 0.2, *p* < 0.001), meaning that turtles increased their swimming effort with the increasing velocity of the oncoming current.

#### Time spent within the plume

The most parsimonious model associated with the lowest AIC contained two variables: the time taken to reach Foraging Grounds (Time to FG) and the minimum FG longitude (closest foraging ground to the nesting site)–see Table 2. There was no relationship between the time spent in Phase 2 and body mass (Spearman correlation test: R^2^ = 0.04, *p* = 0.8626), or swimming speed within the plume (Spearman correlation test: R^2^ = 0.31, *p* = 0.2122). In contrast, Time within the plume increased significantly with the time taken to reach the foraging ground (*p* < 0.01, Table 2), whereas the minimum longitude of foraging grounds decreased significantly with TimePhase2 (*p* < 0.001, Table 2).

**Table 2:** Summary of the linear model performed to relate the TimePhase2* to intrinsic variables.

Variable	Estimate	Std error	Z value	p-value
Time to FG	4.2361	1.5980	2.651	<0.01
Min Longitude FG	-4.9891	1.5980	-3.122	<0.001

TimePhase2* refers to the time (in days) spent within the plume and FG to Foraging Grounds.

#### Diving behavior

**Fig 7:**
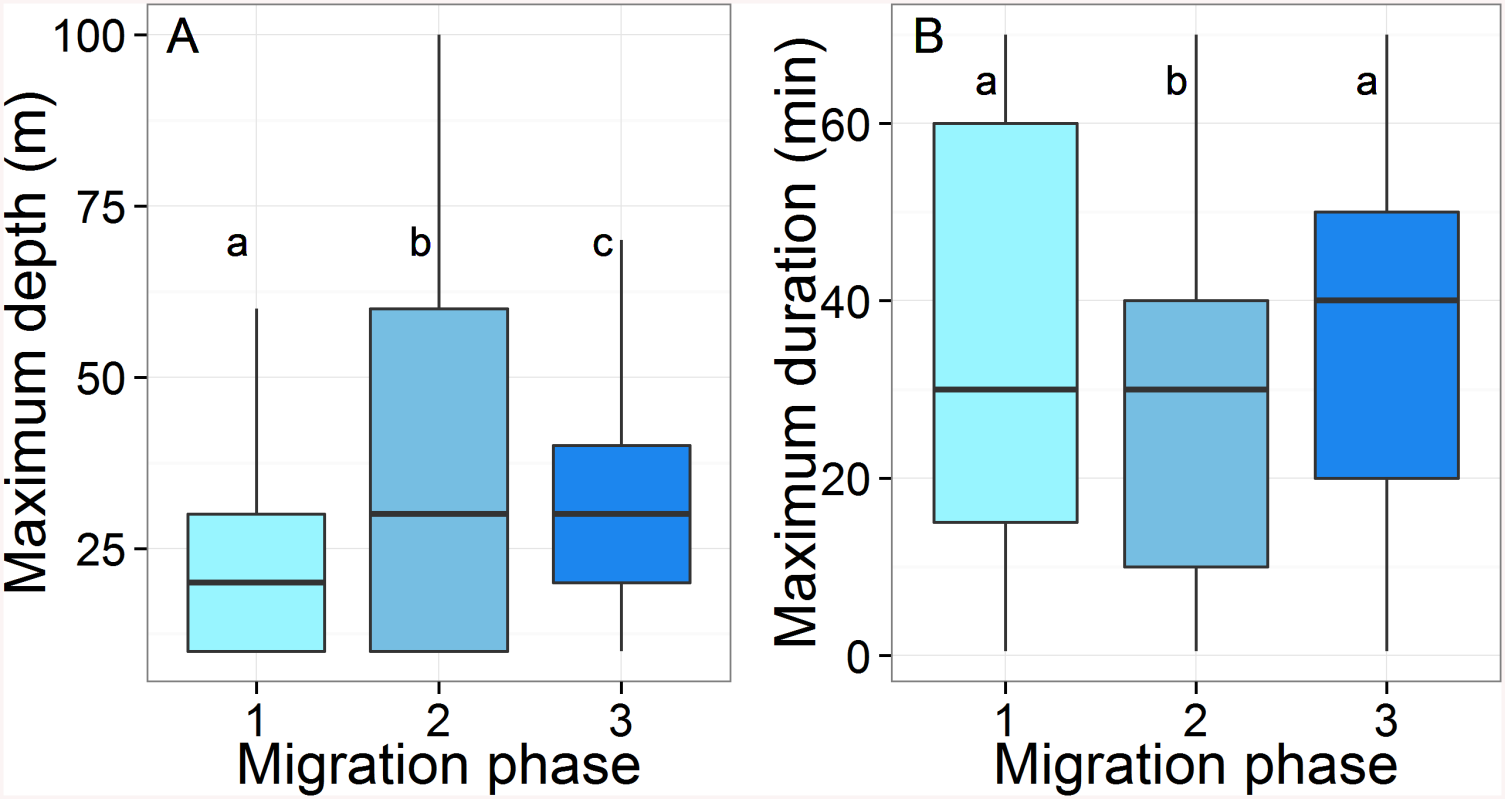
Box plots of (A) the maximum depth (m) and (B) the maximum duration (min) recorded by the 14 Argos-Fastloc GPS tags during the three migration phases. The box plots sharing the same letter are not statistically different (Tukey HDS). The horizontal black line in box plots represents the median.

Mean depth was significantly different between all migration phases (Tukey HSD: *p* < 0.001, Fig 7A), and on average, turtles dived deeper in Phase 2 and to shallower depths in Phase 1 (39.2±21.8 m vs. 26.6±25.3 m, Table 1). Maximum dive duration varied significantly between all migration phases except between Phase 1 and Phase 3 (Tukey HSD: *p* < 0.001, Fig 7B). The longest average dive duration was performed in Phase 3 (37.4±22.5 min), whilst the shortest occurred in Phase 2 (29.1±23.4 min).

## Discussion

Our study uses satellite telemetry and two years of sampling (2012 and 2014) to provide data highlighting the long, counter-current post-nesting migration performed by green turtle *Chelonia mydas* population nesting in French Guiana and Suriname, and reveals a substantial foraging aggregation off the Brazilian coast. The assessment of their spatial and diving patterns in response to environmental variability, i.e. the North Brazil current and the Amazon River plume, provides detailed information on their behavioral adjustments and specific habitat affinities.

### Synchronization and foraging aggregation

A previous study tracked green turtle individuals from the closest rookery to French Guiana, located on the beaches of Tortuguero in Costa Rica [44]. Although there are similarities with our group in terms of coastal migration and proximity to foraging grounds, green turtles from French Guiana and Suriname travelled on average more than three times the maximum distance travelled by the Tortuguero individuals (3681±729 km vs. 1089 km) [44]. Additionally, one individual nesting in French Guiana travelled the longest total distance ever recorded for a green turtle, i.e. 5153 km. This difference in coastal migration distances between the two green turtle populations is striking. Although long travel distances have already been documented for turtles that nest on isolated islands and therefore have to cross the open ocean or the open seas [45,46], they have never been observed before in green turtles migrating exclusively alongshore.

Unlike green turtles in Costa Rica, which displayed migration routes that differed according to individuals [44], the 19 turtles in our study exhibited a remarkable synchronization in time and space since they all travelled in the same direction and during the same period. With the exception of one individual performing a foraging behavior off Cayenne (#115446), the 18 others performed a remarkably straight course to their final destination, and did so within a quite limited time. This pattern suggests an optimal strategy which involves minimizing the cost of migration for turtles by allowing the rapid restoration of their body reserves after the inter-nesting season [45]. The single foraging event located off Cayenne does not correspond to the presence of seagrass beds, and can be attributed to either navigational issues caused by hydrodynamic forcing (this turtle swam in two uniform anticlockwise circles, suggesting that the North Brazil current and/or the north-westward Amazon plume pushed her back in the opposite direction), or a stopover site as identified by Baudouin et al. [21], since this individual could have let itself drift passively in this stopover area.

### Suitable habitat for a mega-herbivore reptile

Our data show that the most favorable habitat conditions for the foraging activity of green turtles are clear and cool waters, which are associated with low bathymetry. Data on seagrass coverage confirms that green turtle foraging grounds are located near seagrass beds. Seagrass is a fragile ecosystem relying on complex biochemical processes, since its growth is regulated by temperature, light and nutrient availability [47,48]. However, the 115.10^7^ tons/year of sediments discharged by the Amazon River plume strongly influence the optical conditions of the north-eastern American coast [24,26], leading to large amounts of suspended and dissolved materials [27] that limit underwater irradiance [47,49]. These unfavorable conditions for seagrass growth may explain why green turtles cross the plume at high travel speeds, i.e. to reach the clearer waters further south that are associated with seagrass meadows. The presence of seagrass beds at green turtle foraging grounds is also noted in the observations of the Federal University of Rio Grande (FURG) [22]. Indeed, five seagrass species have been identified along the tropical coast of Brazil from 0 to 25°S [22]. These species extend over > 30 000 ha, namely *Halodule wrightii*, *H*. *emarginata*, *H*. *decipiens*, *H*. *bailoni* and *Ruppia maritima*. *H*. *wrightii* seems to be the dominant species within green turtle foraging grounds. The biomass of this seagrass species is greater during spring and summer compared to fall and winter [50], which corresponds to the arrival time of green turtles and suggests that the migration timing of this grazer matches the seasonal abundance of seagrass [1].

Ceará was described as a foraging ground for green turtles of the Equatorial Atlantic in the 1970s, when several individuals from French Guiana were observed off the Brazilian coast [51]. Further evidence was seen in 2001 and 2003, when green turtles tagged in Brazil were recaptured in Nicaragua (data available on www.seaturtle.org). Other observations provide links between Ceará foraging grounds and other nesting sites in the tropical Atlantic, such as the Caribbean region and Central America. Furthermore, green turtles nesting on Ascension Island migrate to foraging grounds off Paraíba state [51,52], a site reached by one of the individuals in our study (#115460).

The turtles performed longer dives in foraging areas, which strongly suggests the occurrence of feeding activity [10,44]. The difference in water temperature within the plume and inside foraging grounds (~1.9°C) can be explained by the warmer freshwater supply from the Amazon River [53]. Cooler temperatures in foraging areas may therefore play two roles: firstly they favor seagrass development under optimal conditions [48], and secondly they allow these ectothermic organisms to reduce their metabolism and thus minimize their energy expenditure [54]. A reduced metabolic rate may therefore enable turtles to dive for longer periods, optimizing resource exploitation [55].

### Dispersal adaptations to counterbalance the effect of strong currents

The migration trajectories of these 19 green turtles highlight a counter-current migration, with inter-individual variability observed for the distance to shore and vertical movements. The surface current velocities show that turtles swim against the current throughout their migration to foraging grounds, which could require high amounts of energy. All individuals travelled at high speeds to compensate for current-induced drift, and approached the speeds reached by green turtles crossing the Atlantic Ocean from Ascension Island (0.56 m.s^-1^ vs. 0.71 m.s^-1^) [21]. However, no documentation to date describes the swimming speed of green turtles after correction for currents. This study provides the first reliable data ever recorded for swimming speeds, and reveals that individuals reached on average 0.84 m.s^-1^, with bursts of speed attaining 3.50 m.s^-1^. It is important to note that higher swimming speeds were recorded at the beginning of the migration, where the current velocities are the strongest, i.e. the Guiana current. Consequently, green turtles could either increase their swimming speed in response to current velocity, i.e. an increase of 46% compared to the initial observed speed, or simply travel faster before the plume because they have more energy at the beginning of the migration than at the end of their journey, thousands of kilometers away.

The North Brazil current carries upper-ocean waters northwards to the Equator with a maximum transport of up to 36 Sv (Sv = 10^6^ m^3^ s^-1^) at depths of over 600 m [56]. By keeping their trajectories confined close to the shore, i.e. within an average 23 km from the coastline, turtles show a strategy to save energy by avoiding the strong North Brazil current, which flows at its highest velocities at around 100 km offshore. Furthermore, the post-nesting migration of green turtles spans from April to September; this period coincides with the velocity peak of the North Brazil current, which is at its highest from July to August [56]. Despite the great speed of the North Brazil current, turtles may also be affected by tidal processes as they are travelling very close to the shore. Over the French Guiana continental shelf, tidal currents therefore influence the inner shelf, within 15–20 km from the coastline [57]. During flood of spring tides, tidal currents are directed to the coast, and thus influence the total currents, reaching up to 0.45 m.s^-1^ [57]. The alongshore current can therefore be turned into a cross-shore current, that favor turtles displacements. Decreasing bathymetry combined with bottom friction may also play a significant role in coastal current dissipation alongshore [58,59], explaining why turtles keep their trajectories close to the shore before and after the plume, saving energy during migration. In addition, the first individuals to leave the nesting site in April probably avoided the peak strength of the North Brazil current, giving them the opportunity to reach their foraging grounds rapidly and begin their foraging activity further south. In contrast, turtles would face higher energy requirements when migrating at the end of the season, i.e. when the currents are at their strongest at the end of May or even during June.

### Behavioral adjustments to cross the Amazon River plume

The significant increase in distance to shore for all individuals when crossing the plume indicates that the green turtles also adapted their behavior to the Amazon River plume. Two different spatial patterns appeared: some individuals remained close to shore during the plume crossing, whereas others swam farther from the shore, i.e. up to 200 km offshore. The latter spent more time in the plume phase due to the higher current velocity at greater distances from the coastline. This suggests that travelling alongshore when crossing the plume is probably the optimal dispersal strategy to avoid the strong North Brazil current and reach the foraging grounds more rapidly for body reserve repletion. However, turtles could also cross the mouth of the Amazon further out to sea, either to avoid the high current velocity of the plume, or, more probably, to reduce the distance travelled by choosing the shortest path to reach the foraging grounds instead of following the coastline [10].

Although the strength of the plume appears to be relatively stable across the years, the Amazon River plume shows a notable seasonal variability [53,60,61], with river discharges that attain maximum levels of approximately 2.4 x 10^5^ m^3^ s^-1^ between May and June [53]. This variability could lead to the selection of different foraging areas within the breeding seasons, with a potential lack of site fidelity among individuals. Indeed, unlike the green turtles of the Mediterranean sea, which use similar migratory routes to reach the same foraging grounds from one year to the next [62], green turtles in the Equatorial Atlantic may use different foraging areas off the Brazilian coast throughout the breeding seasons, depending on the date migration begins. Indeed, the interannual variability of the number of clutches laid and the variable remigration interval (from 2 to 3 years) observed in green turtles of the Mediterranean Sea [63] could potentially affect the duration of the inter-nesting season, and consequently the month migration starts. In turn, it might define the month during which turtles might cross the river plume. It would therefore be interesting in a future study to tag the same individuals to compare their different post-nesting migration routes and assess site fidelity.

Crossing this physical barrier may alter the rest of the migration, and consequently affect the choice of foraging ground location and the time required to reach foraging grounds. Our data show that the longer individuals stayed within the plume, the sooner they initiated foraging on seagrass beds, suggesting that the crossing of the highly turbulent plume resulted in higher energy expenditure. In contrast, turtles that crossed the plume at a higher speed stopped further south, where seagrass beds tend to be of higher quality and are more abundant. The date of departure was an important factor, as the first individuals to leave the nesting site crossed the plume more rapidly. Indeed, given that the velocity peak of the Amazon plume is reached in May-June, individuals that left French Guiana early in the inter-nesting season, i.e. in April or beginning of May probably avoided the peak of water discharges, therefore limiting their energy expenditure. Nevertheless, whatever the time spent within the plume, travel speed was higher for all individuals within the plume, confirming the need to cross this unfavorable area (without seagrass or visibility) as fast as possible, as there is no possibility to feed en-route [10]. After the plume, the reduced travel speeds associated with two different stopover areas identified by Baudouin et al. [21] reinforce the assumption that individuals would need to recover after crossing such a turbulent zone.

Diving behavior might also change during migration in an effort to adapt to various abiotic conditions. Simulations of the Amazon River discharge have demonstrated a weak return current in the underlying seawater beneath the surface and a reduction of the plume velocity in deeper layers [53]. Since the plume is 3–10 m deep [61], individuals could avoid the strong current and turbid surface layer by targeting specific depths beyond 10 m. This could be confirmed by retrieving the tags and downloading the high resolution data that were not transmitted. The shorter dives within the plume highlight the energetic costs involved in crossing such a turbulent area, and could be a means to avoid being swept seaward by the Amazon flow. Short dives could also enable green turtles to return to the surface more frequently, as they may use airborne odorants associated with land to maintain their course in the highly turbid waters of the plume. This theory was demonstrated in loggerhead turtles [64] and could be an additional orientation cue used by green turtles nesting in French Guiana during their migration. It would be interesting to deploy acceleration data loggers in a further study to determine the relationships between energetic expenditure and environmental variables and investigate whether green turtles feed en-route before reaching their foraging grounds [65,66].

## Conclusions and Perspectives

Our data provide detailed information on the habitat requirements for one of the two main activities occurring in the life cycle of adult green turtles, i.e. post-nesting migration to foraging areas. This study highlights several behavioral adjustments in both horizontal and vertical movements in response to a highly dynamic zone under the influence of the North Brazil Current and the Amazon River plume. Unlike green turtles from Ascension Island, which use the South Atlantic equatorial current during their post-nesting migration [67], green turtles from French Guiana seem to perform the opposite strategy and swim against the currents during their post-nesting migration. The deployment of tags on females at the foraging grounds in Brazil, before their return journey to French Guiana, would be an opportunity to investigate the potential use of the North Brazil current by green turtles on their return trip to optimize the energy stores they have gained at the foraging grounds. Contrary to the pattern seen over their migration towards the foraging grounds, turtles might therefore migrate farther from the coast on their return journey in order to take advantage of stronger currents.

## Supporting Information

S1 Fig **a) Variances of the FPT according to the ARS spatial scale (*r* in km) for each individual**. The red dotted lines and the bold numbers indicate the radii referring to the highest FPT variances. **b) FPT (in days) over time for the optimum radii of each individual**. The red lines indicate Lavielle segmentation corresponding to the ARS events.
(DOCX)

S2 Fig Monthly K_d_ distributions (in m^-1^) over the whole study area extracted daily from Mercator-Ocean GLORYS-2v1 (Global Ocean ReanalYsis and Simulations) in (A) April, (B) May, (C) June, (D) July, (E) August, (F) September and (G) October. The shoreline was extracted from NOAA National Geophysical Data Center, Coastline.
(DOCX)

S1 Table Summary of the horizontal movements of the 19 individuals over the entire tracking period. Nloc refers to the total number of positions recorded per individual.
(DOCX)
