# Supplementary material for: Dispersal and Diving Adjustments of the Green Turtle Chelonia mydas in Response to Dynamic Environmental Conditions during Post-Nesting Migration
Source: PLoS One. 2015 Sep 23;10(9):e0137340. doi: 10.1371/journal.pone.0137340 (PMC4580322; doi:10.1371/journal.pone.0137340)

**Supporting information S1**


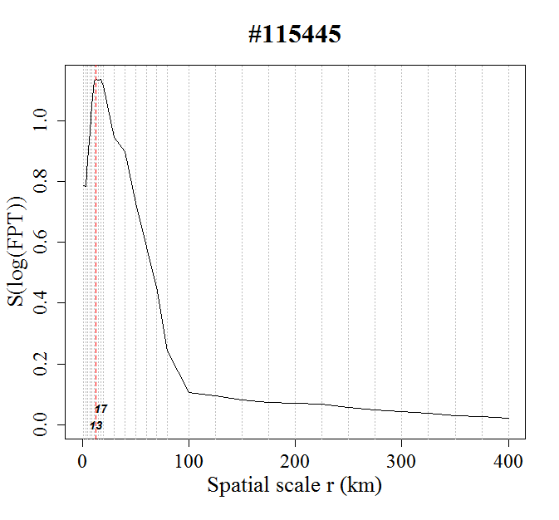

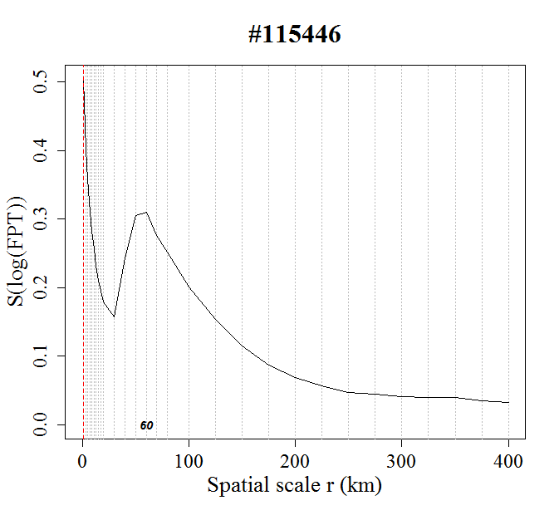

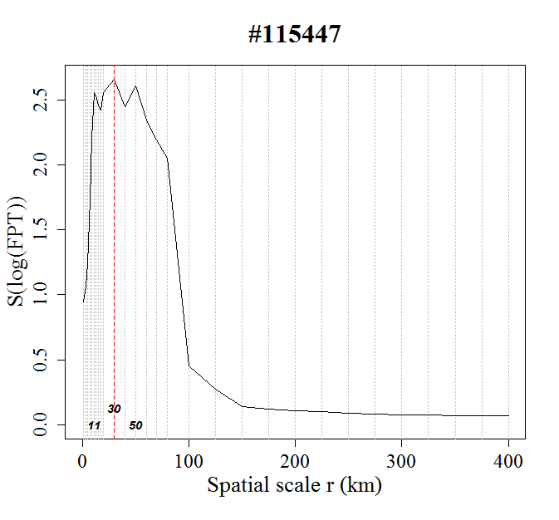

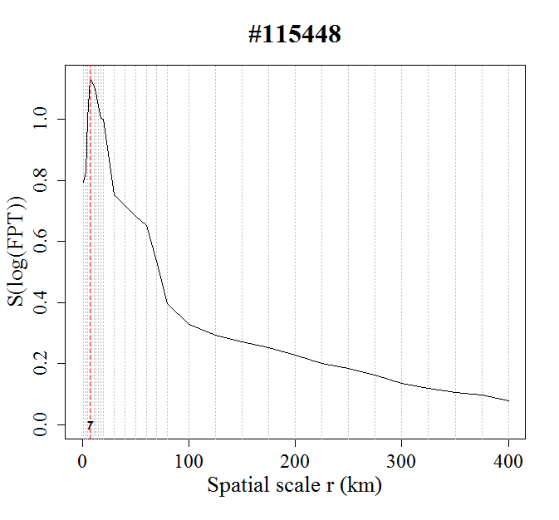

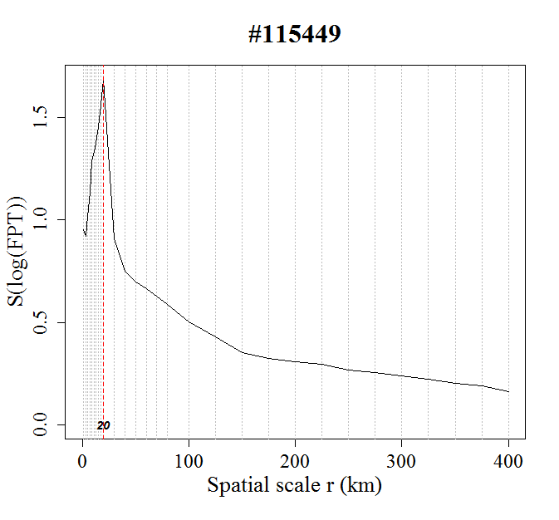

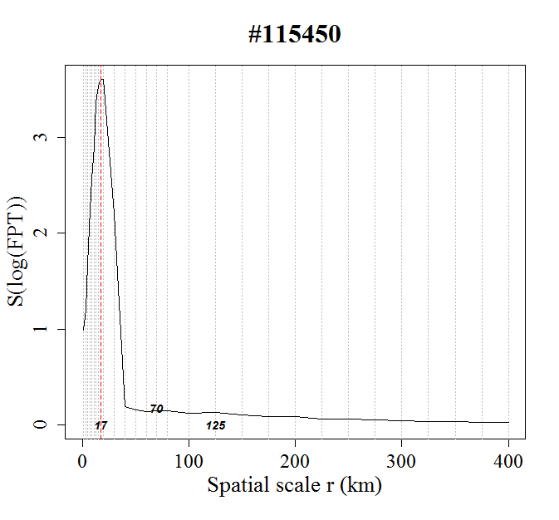

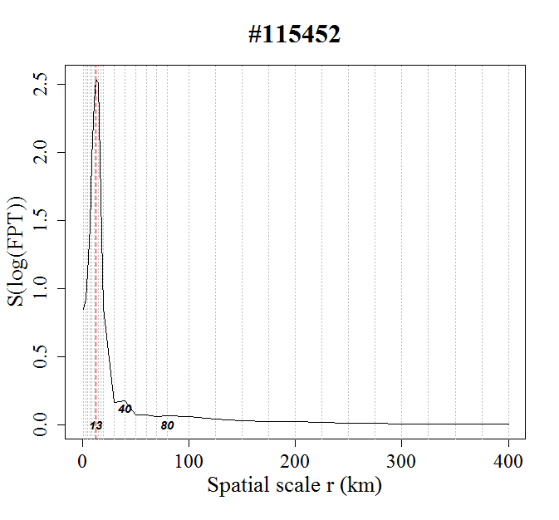

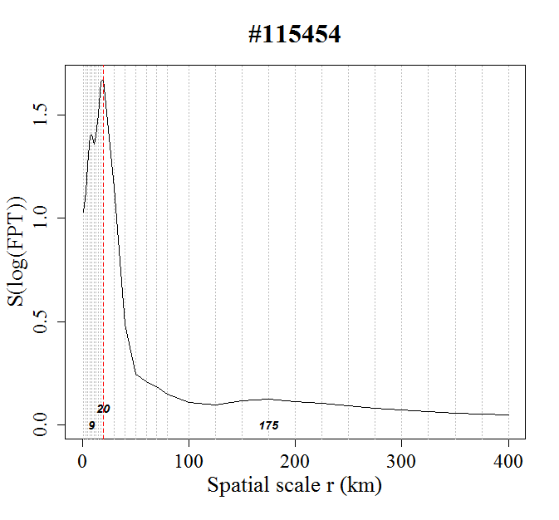

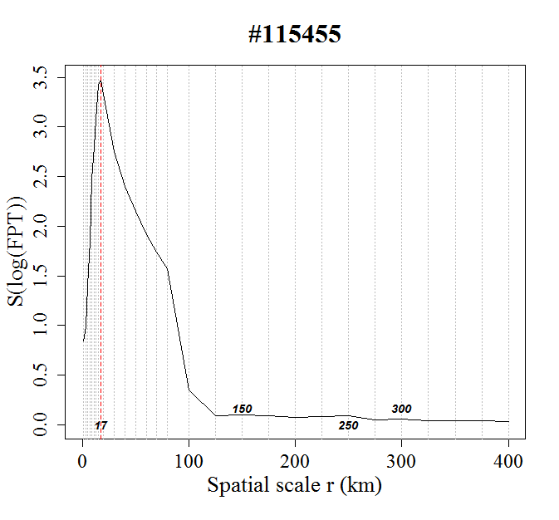

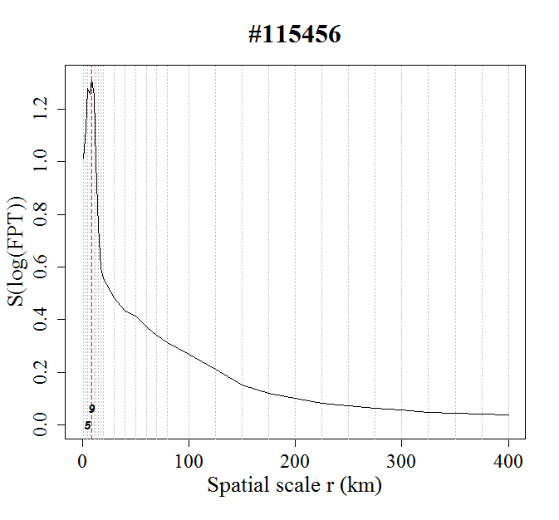

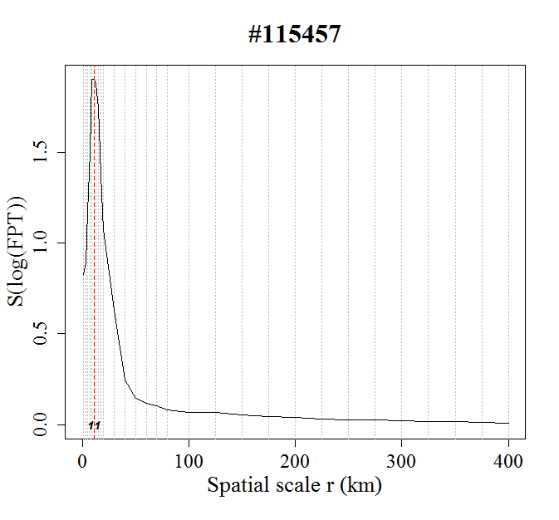

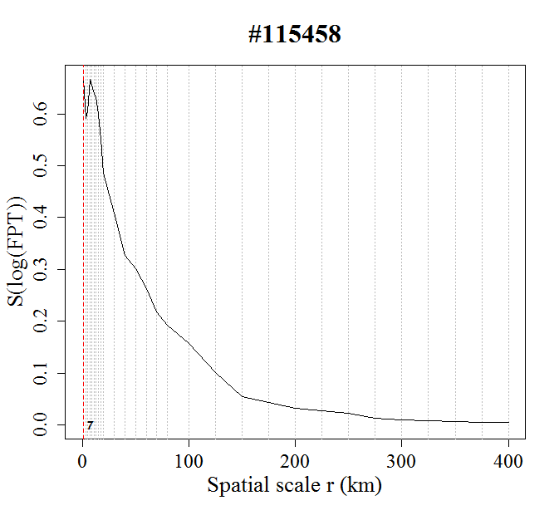

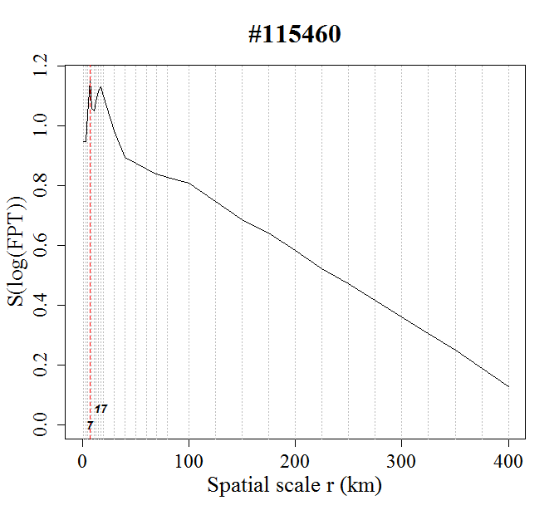

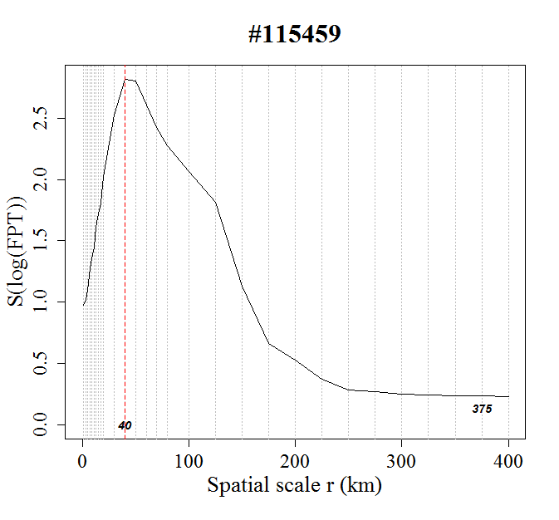

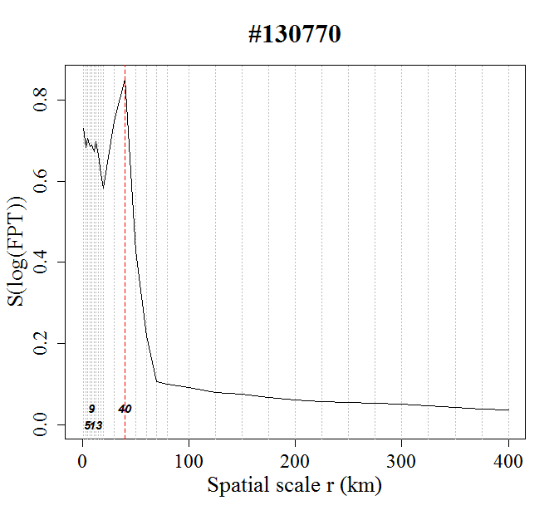

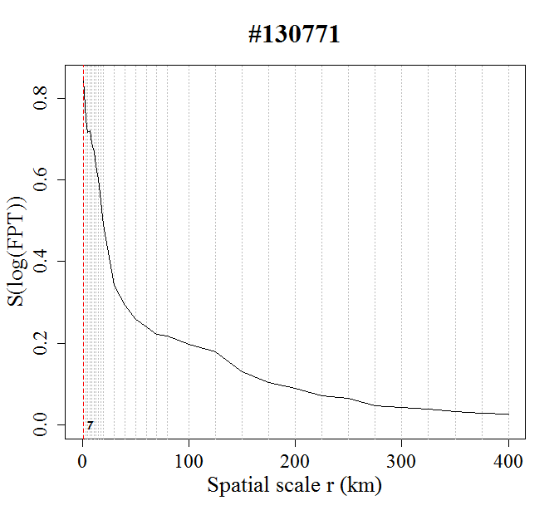

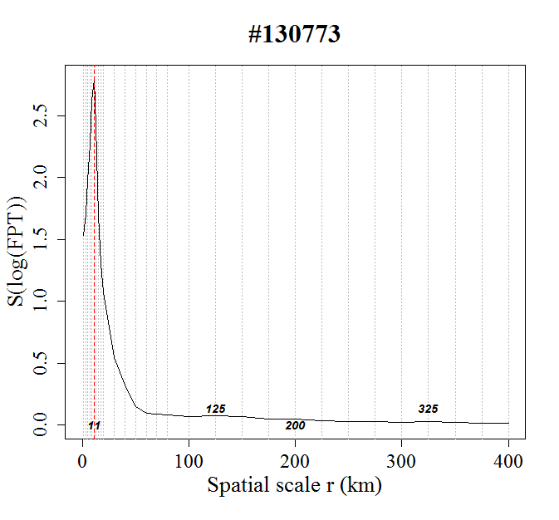

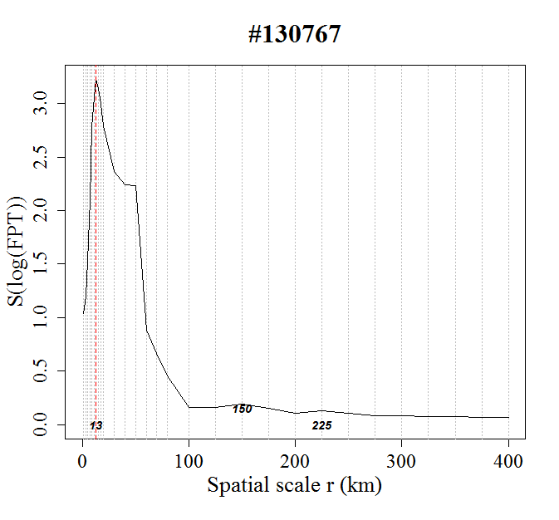

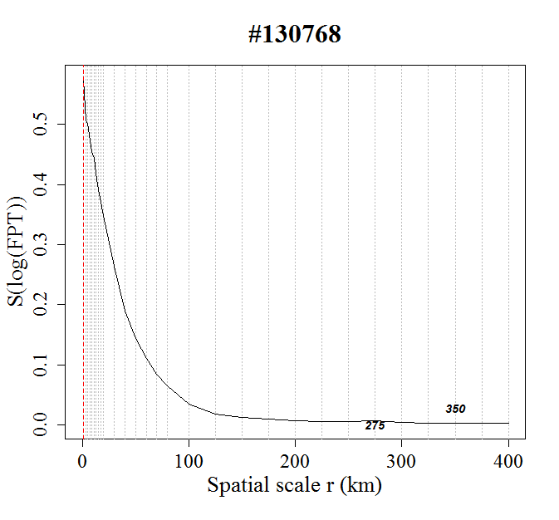


**S1a Figure. Variances of the FPT according to the ARS spatial scale (r in km) for each individual.** The red dotted lines and the bold numbers indicate the radii referring to the highest FPT variances.


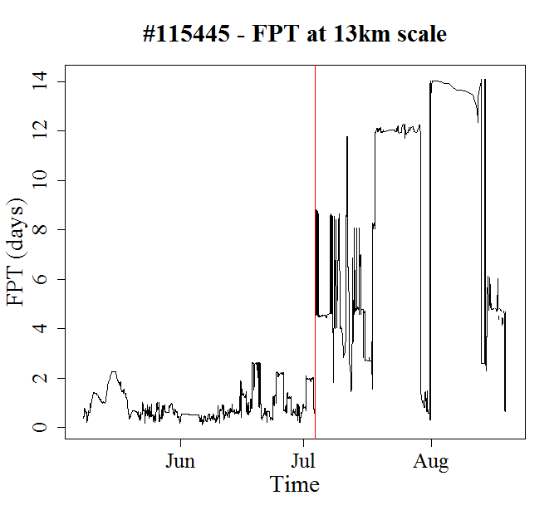

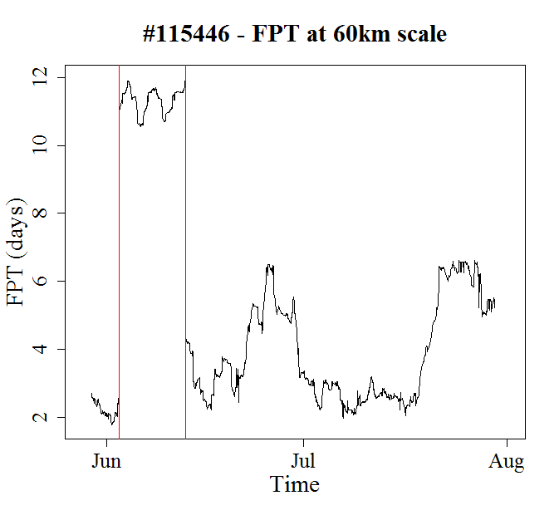

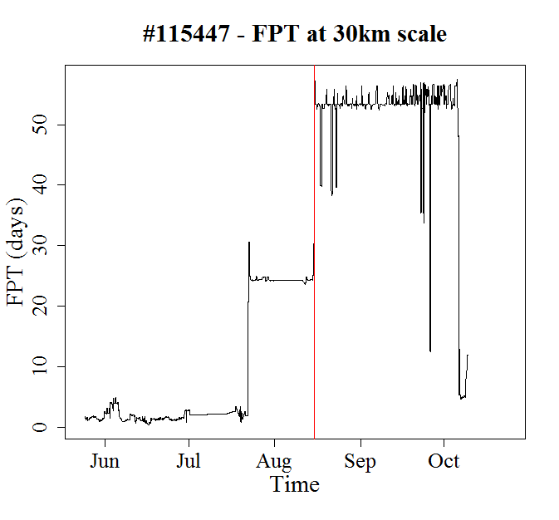

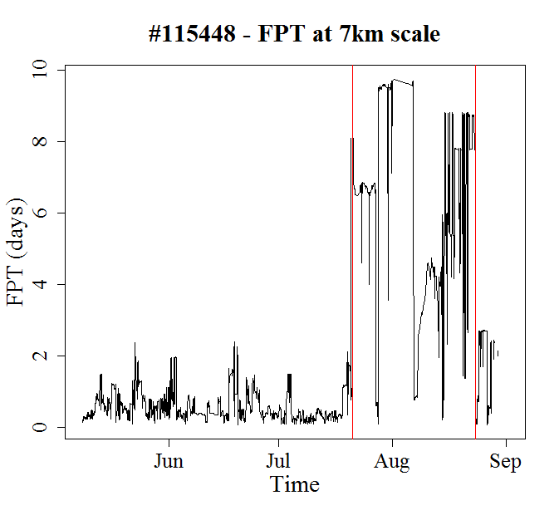

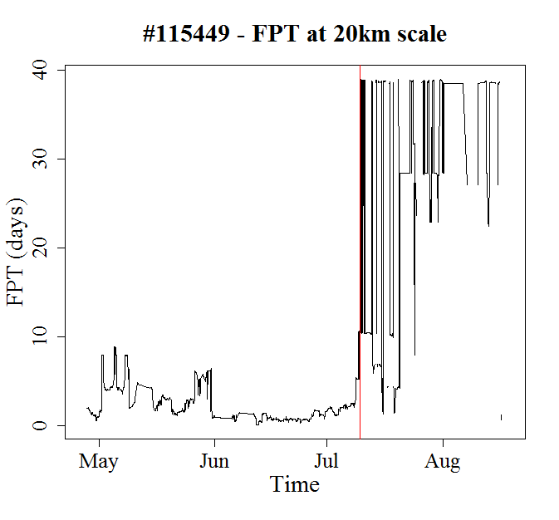

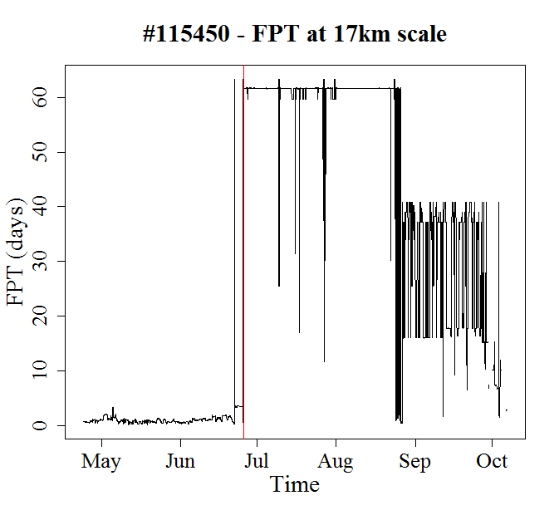

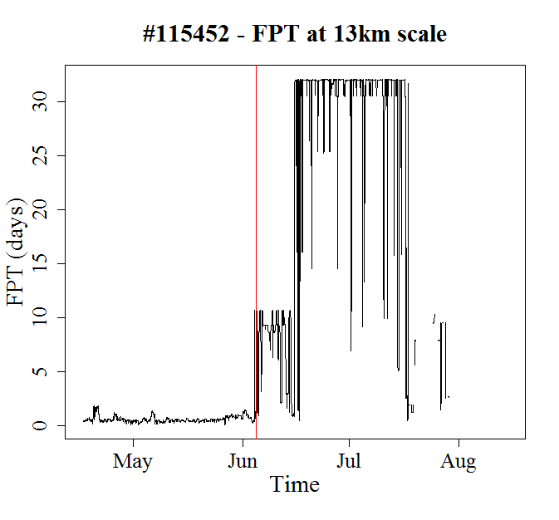

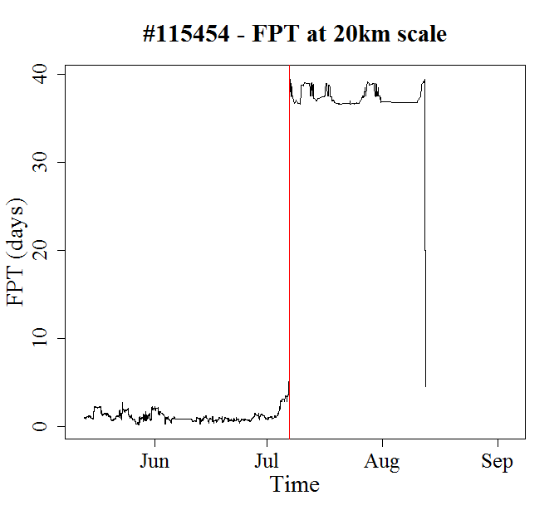

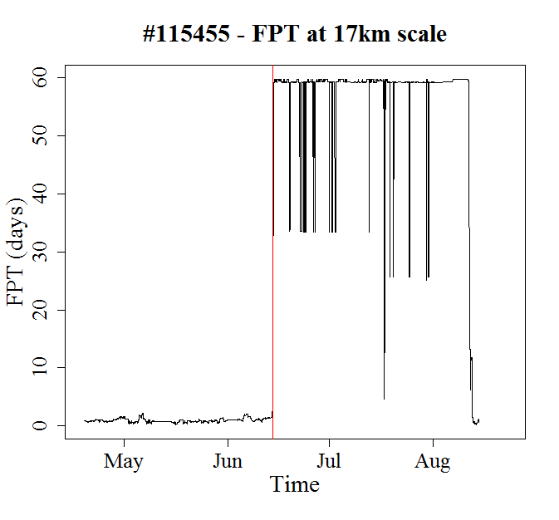

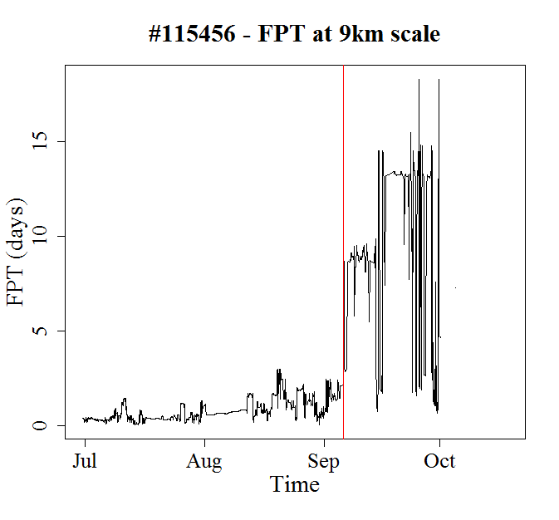

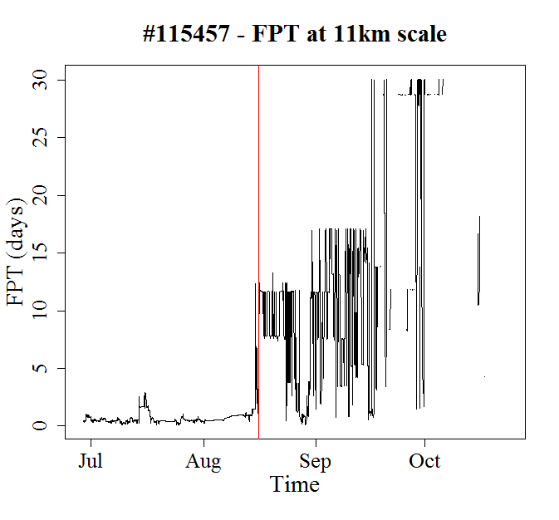

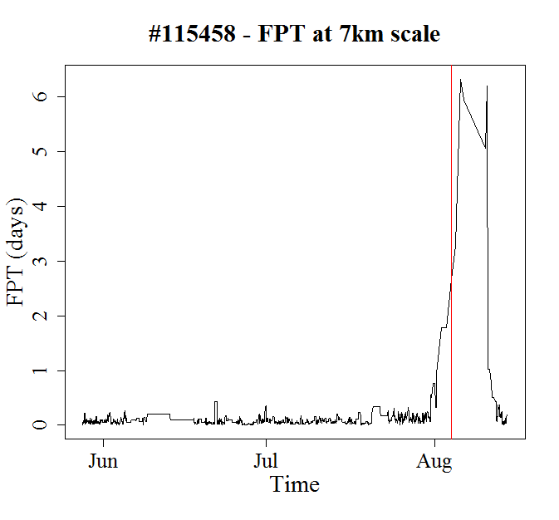

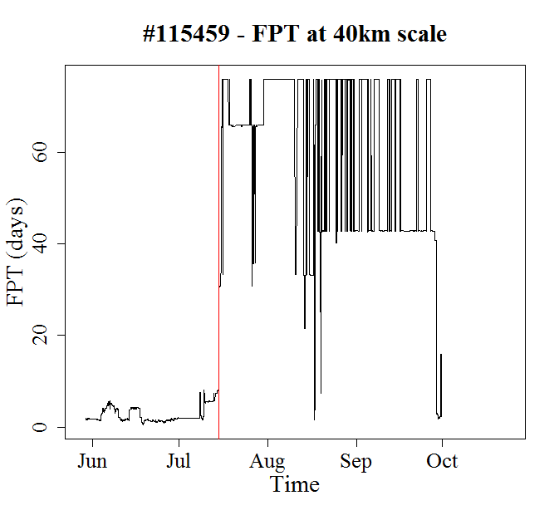

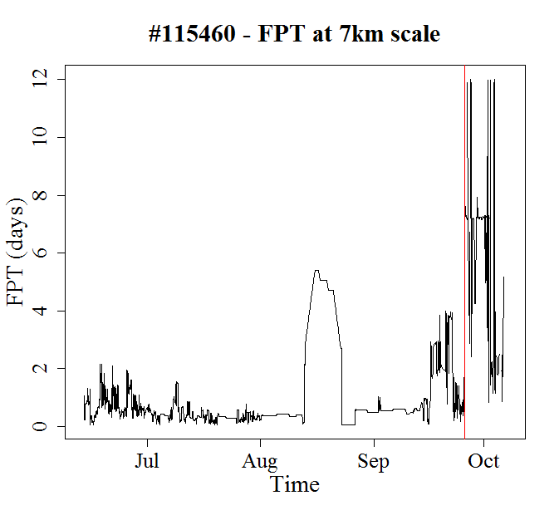


**S1b Figure. FPT (in days) over time for the optimum radii of each individual.** The red lines indicate Lavielle segmentation corresponding to the ARS events.


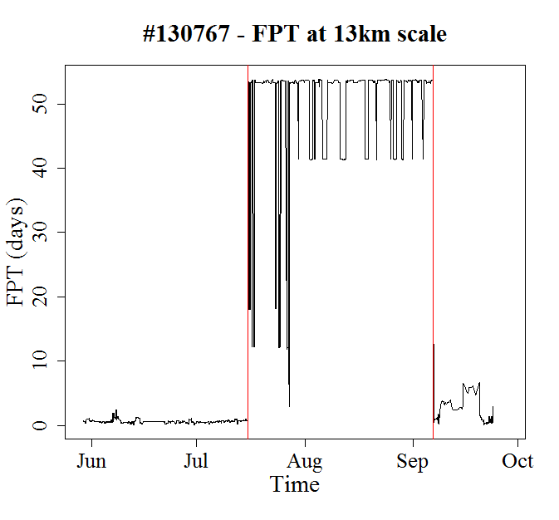

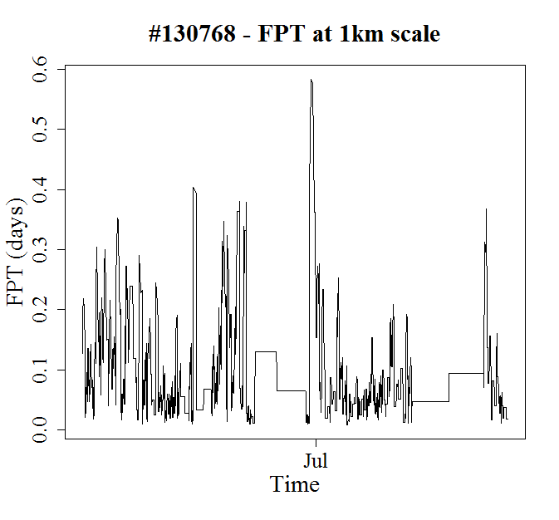

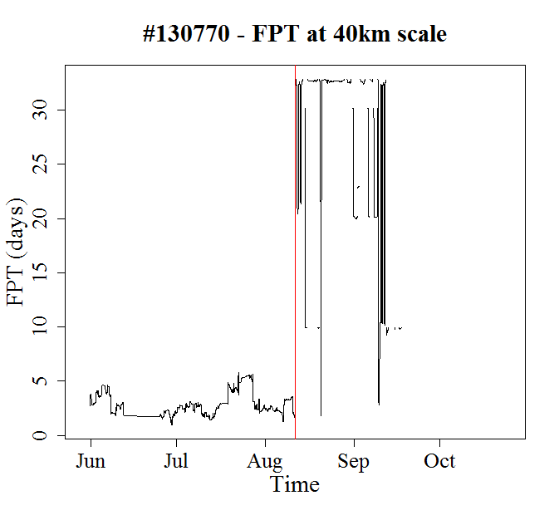

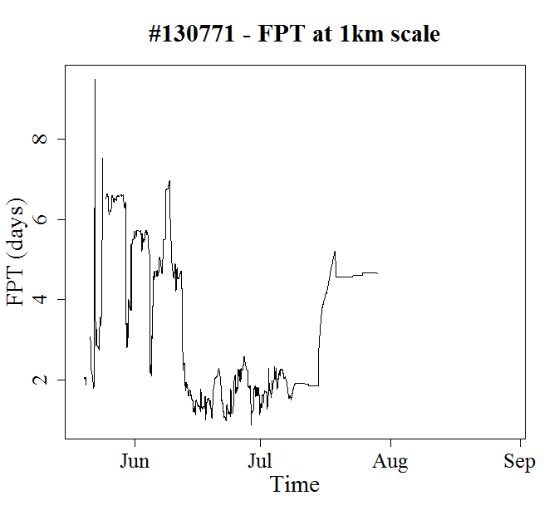

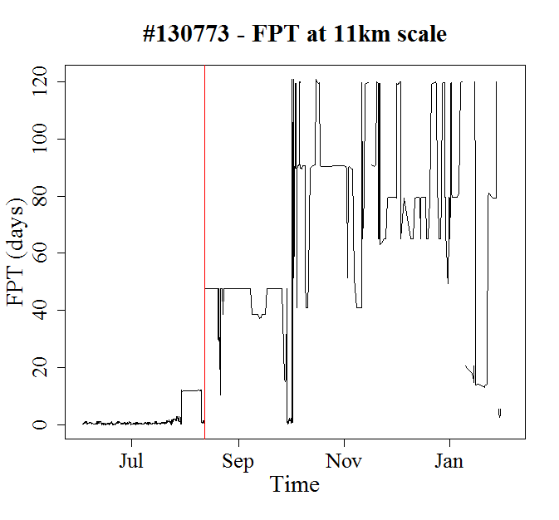

Supplement: S1 Fig — a) Variances of the FPT according to the ARS spatial scale (r in km) for each individual. The red dotted lines and the bold numbers indicate the radii referring to the highest FPT variances. b) FPT (in days) over time for the optimum radii of each individual. The red lines indicate Lavielle segmentation corresponding to the ARS events. (DOCX) [file pone.0137340.s001.docx]
