# Supplementary material for: Dispersal and Diving Adjustments of the Green Turtle Chelonia mydas in Response to Dynamic Environmental Conditions during Post-Nesting Migration
Source: PLoS One. 2015 Sep 23;10(9):e0137340. doi: 10.1371/journal.pone.0137340 (PMC4580322; doi:10.1371/journal.pone.0137340)
Supplement: S2 Fig — The shoreline was extracted from NOAA National Geophysical Data Center, Coastline. (DOCX) [file pone.0137340.s002.docx]

# Supporting information S2


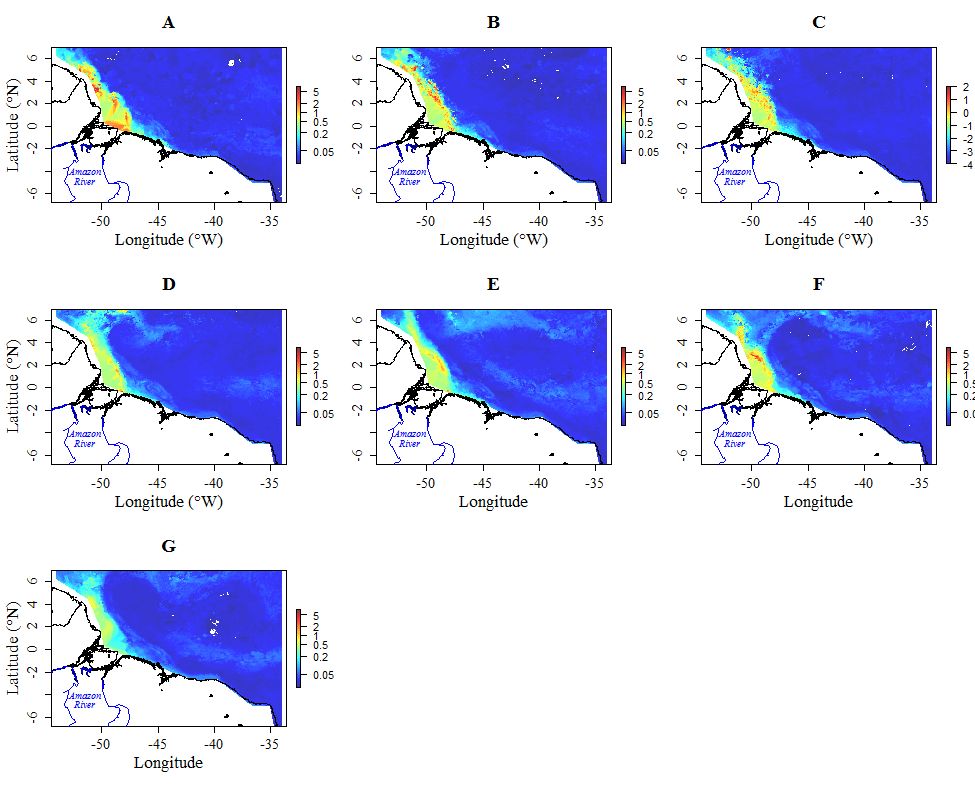


**S2 Figure 1. Monthly K_d_ distributions (in m^-1^) over the whole study area in (A) April, (B) May, (C) June, (D) July, (E) August, (F) September and (G) October.**
