# Supplementary material for: Dispersal and Diving Adjustments of the Green Turtle Chelonia mydas in Response to Dynamic Environmental Conditions during Post-Nesting Migration
Source: PLoS One. 2015 Sep 23;10(9):e0137340. doi: 10.1371/journal.pone.0137340 (PMC4580322; doi:10.1371/journal.pone.0137340)
Supplement: S1 Table — Nloc refers to the total number of positions recorded per individual. (DOCX) [file pone.0137340.s003.docx]

**Supporting Information S1**

**S1 Table. Summary of the horizontal movements of the 19 individuals over the entire tracking period.** Nloc refers to the total number of positions recorded per individual.

| **Ptt** | **Tag type** | **Start Date** | **End Date** | **Arrival date in ARS** | **Nloc** | **CCL** | **Mass** | **Tracking duration** | **Distance travelled** | **Observed speed** | **Swimming speed** | **ARS scale** |
| --- | --- | --- | --- | --- | --- | --- | --- | --- | --- | --- | --- | --- |
|  |  |  |  |  |  | **(cm)** | **(kg)** | **(d)** | **(km)** | **(m.s-^1^)** | **(m.s-^1^)** | **(km)** |
| 115445 | MK10 | 08/05/2012 | 19/08/2012 | 04/07/2012 | 665 | 115 | 173.2 | 103 | 3517 | 0.63±0.56 | 0.81±0.56 | 13 |
| 115446 | MK10 | 28/05/2012 | 01/08/2012 | 03/06/2012 | 548 | 113 | 165.7 | 65 | 2971 | 0.70±0.53 | 1.10±0.58 | 60 |
| 115447 | MK10 | 23/05/2012 | 24/10/2012 | 23/07/2012 | 916 | 114 | 169.5 | 154 | 4110 | 0.48±0.52 | 0.71±0.52 | 30 |
| 115448 | MK10 | 08/05/2012 | 01/09/2012 | 21/07/2012 | 892 | 119 | 188.2 | 116 | 3989 | 0.62±0.57 | 0.93±0.60 | 7 |
| 115449 | MK10 | 26/04/2012 | 18/08/2012 | 09/07/2012 | 633 | 120 | 192 | 114 | 3030 | 0.51±0.51 | 0.84±0.50 | 20 |
| 115450 | MK10 | 23/04/2012 | 07/10/2012 | 25/06/2012 | 1415 | 117 | 180.7 | 167 | 4932 | 0.55±0.55 | 0.84±0.55 | 17 |
| 115452 | MK10 | 16/04/2012 | 14/08/2012 | 04/06/2012 | 938 | 117 | 180.7 | 120 | 4013 | 0.66±0.57 | 0.90±0.57 | 13 |
| 115454 | MK10 | 12/05/2012 | 03/09/2012 | 07/07/2012 | 800 | 107 | 143.2 | 114 | 3393 | 0.60±0.59 | 0.85±0.55 | 20 |
| 115455 | MK10 | 18/04/2012 | 16/08/2012 | 14/06/2012 | 1111 | 123 | 203.2 | 120 | 3835 | 0.54±0.53 | 0.76±0.53 | 17 |
| 115456 | MK10 | 30/06/2012 | 18/10/2012 | 07/09/2012 | 753 | 111 | 158.2 | 110 | 3245 | 0.53±0.56 | 0.82±0.57 | 9 |
| 115457 | MK10 | 29/06/2012 | 23/10/2012 | 16/08/2012 | 786 | 118 | 184.5 | 116 | 3321 | 0.58±0.54 | 0.79±0.53 | 11 |
| 115458 | MK10 | 28/05/2012 | 14/08/2012 | 06/08/2012 | 292 | 110 | 154.5 | 78 | 2816 | 0.63±0.51 | 0.91±0.49 | 7 |
| 115459 | MK10 | 28/05/2012 | 23/10/2012 | 15/07/2012 | 998 | 113 | 165.7 | 148 | 4366 | 0.59±0.58 | 0.82±0.59 | 40 |
| 115460 | MK10 | 13/06/2012 | 07/10/2012 | 25/09/2012 | 644 | 116 | 177 | 116 | 4163 | 0.62±0.57 | 0.90±0.55 | 7 |
| 130767 | CTD-SRDL | 29/05/2014 | 31/08/2014 | 15/07/2014 | 562 | 116 | 177 | 94 | 2781 | 0.48±0.51 | 0.72±0.49 | 13 |
| 130768 | CTD-SRDL | 04/06/2014 | 22/07/2014 | - | 169 | 120 | 192 | 48 | 2055 | 0.73±0.59 | 0.94±0.62 | - |
| 130770 | CTD-SRDL | 28/05/2014 | 24/10/2014 | 11/08/2014 | 357 | 115 | 173.2 | 149 | 3427 | 0.53±0.48 | 0.78±0.50 | 40 |
| 130771 | CTD-SRDL | 19/05/2014 | 28/08/2014 | - | 281 | 113 | 165.7 | 101 | 2825 | 0.68±0.59 | 0.97±0.57 | - |
| 130773 | CTD-SRDL | 02/06/2014 | 31/12/2014 | 15/08/2014 | 377 | 133 | 240.7 | 212 | 2773 | 0.58±0.53 | 0.92±0.52 | 11 |
|  |  |  |  |  | 691±318 | 116.3±5.5 | 188.2±8.6 | 118±37 | 3450±701 | 0.58±0.55 | 0.84±0.55 | 19.7±14.6 |
